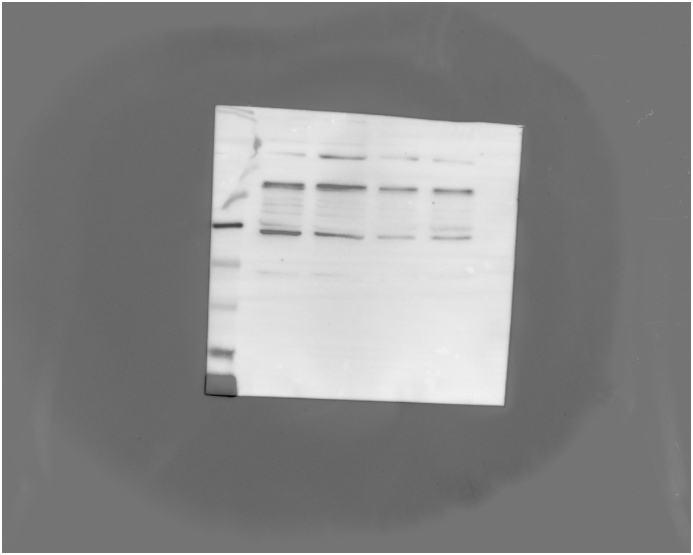# Caveolin-1 promotes glioma proliferation and metastasis by enhancing EMT via mediating PAI-1 activation and its correlation with immune infiltrates

**DOI:** 10.1016/j.heliyon.2024.e24464

**Published:** 2024-01-14

**Authors:** Zhaoxiang Wang, Gang Chen, Debin Yuan, Peizhang Wu, Jun Guo, Yisheng Lu, Zhenyu Wang

**Affiliations:** a Department of Neurosurgery, Yancheng First Hospital, Affiliated Hospital of Nanjing University Medical School, No. 166 Yulong West Road, Yancheng, 224000, Jiangsu, China; b Department of Neurosurgery, The First People's Hospital of Yancheng, No. 166 Yulong West Road, Yancheng, 224000, Jiangsu, China; c Department of Neurosurgery, Affiliated Hospital of Nantong University, Nantong University, Jiangsu, 226001, China; d Department of Pediatric General Surgery, Shanghai Children's Hospital, School of Medicine, Shanghai Jiaotong University, No. 355 Luding Road, Shanghai, 200062, Shanghai, China

**Keywords:** CAV-1, PAI-1, Glioma, EMT, Metastasis, Immune infiltrates

## Abstract

Glioma is typically characterized by a poor prognosis and is associated with a decline in the quality of life as the disease advances. However, the development of effective therapies for glioma has been inadequate. Caveolin-1 (CAV-1) is a membrane protein that plays a role in caveolae formation and interacts with numerous signaling proteins, compartmentalizing them in caveolae and frequently exerting direct control over their activity through binding to its scaffolding domain. Although CAV-1 is a vital regulator of tumour progression, its role in glioma remains unclear. Our findings indicated that the knockdown of CAV-1 significantly inhibits the proliferation and metastasis of glioma. Subsequent mechanistic investigations demonstrated that CAV-1 promotes proliferation and metastasis by activating the photoshatidylinositol 3-kinase/protein kinase B (PI3K/Akt) signaling pathway. Furthermore, we demonstrated that CAV-1 overexpression upregulates the expression of serpin peptidase inhibitor, class E, member 1 (SERPINE1, also known as PAI-1), which serves as a marker for the epithelial-mesenchymal transition (EMT) process. Further research showed that PAI-1 knockdown abolished the CAV-1 mediated activation of PI3K/Akt signaling pathway. In glioma tissues, CAV-1 expression exhibited a correlation with unfavorable prognosis and immune infiltration among glioma patients. In summary, our study provided evidence that CAV-1 activates the PI3K/Akt signaling pathway by upregulating PAI-1, thereby promoting the proliferation and metastasis of glioma through enhanced epithelial-mesenchymal transition (EMT) and angiogenesis, and CAV-1 is involved in the immune infiltration.

## 1 Introduction

Glioma, originating from neuroepithelial cells, is a prevalent malignant tumor of the central nervous system. Currently, clinical treatment mainly relies on chemotherapy and surgery [1,2]. However, due to the high invasiveness of glioma, patients exhibit a high recurrence rate after surgery [3,4]. Tumor metastasis is an important cause of poor prognosis in glioma patients. In recent years, targeted therapy has emerged as a promising approach to enhance the survival rate of glioma patients [5,6]. Therefore, there is an urgent need to study the molecular mechanisms underlying glioma metastasis and elucidate the targets influencing glioma progression. This research may lead to the development of novel strategies for targeted glioma treatment. EMT is a cellular process in which epithelial cells obtain their mesenchymal phenotype through specific changes in gene expression [7]. During this process, epithelial cells acquire the ability to migrate and invade [8]. Numerous studies have demonstrated the necessity of EMT for tumor cells to reduce cell-to-cell adhesion and acquire motility [9]. Therefore, EMT is recognized as an important process that facilitates tumor cell invasion and malignant tumor progression [10,11]. EMT induces changes in epithelial plasticity, primarily characterized by decreased expression of epithelial markers like E-cadherin and increased expression of mesenchymal proteins such as N-cadherin and Vimentin [12]. Recent studies have shown that EMT is involved in the metastasis and recurrence of tumor cells in glioma. EMT is also associated with chemotherapy resistance and plays a crucial role in the treatment response of gliomas. Consequently, targeting EMT to inhibit metastasis and enhance chemotherapy sensitivity holds promise as a strategy for glioma treatment. Angiogenesis plays an important role in the cancer development, promoting the development and metastasis of tumors by providing oxygen and nutrients [13,14]. Fokkman demonstrated that tumor development relies on the vital process of angiogenesis [15], suggesting that obstructing tumor blood supply could lead to cancer eradication. Therefore, exploring the potential mechanisms that regulate tumor angiogenesis is of great significance for the treatment of tumors, which may provide a more effective approach for treating tumor metastasis.

Caveolin-1 (CAV-1) is a regulatory protein located in the foveal region of the cell membrane, playing a crucial role in the formation of plasma membrane invaginations known as caveolae [16]. In the fovea, CAV-1 interacts with various signal molecules, including G protein coupled receptors, tyrosine kinases and GTPases. Currently, several studies have shown that the overexpression of CAV-1 is related to cancer progression, angiogenesis and metastasis [17,18]. Some studies have found that CAV-1 has the potential to alter the molecular basis underlying the pathobiology of brain tumors, particularly the malignant glial subtype [19]. However, the molecular mechanism and functional effects of CAV-1 in glioma are still elusive.

In this study, we found that CAV-1 was upregulated in glioma. Overexpression of CAV-1 promoted the metastatic potential of glioma cells. Molecular and functional experiments indicated that CAV-1 upregulates PAI-1 expression, activates the PI3K/Akt signaling pathway, and promotes EMT and angiogenesis in glioma cells. Moreover, overexpression of CAV-1 was positively correlated with immune cell infiltration levels of macrophage cells, neutrophils and immature dendritic cells and negatively correlated with the infiltration levels of plasmacytoid dendritic cells, NK CD56bright cells and Follicular helper T cell. In summary, our research findings demonstrate that overexpression of CAV-1 activates the PI3K/Akt signaling pathway by mediating upregulation of PAI-1, ultimately promoting glioma metastasis. CAV-1 has the potential to serve as a prognostic factor and therapeutic target for glioma.

## 2 Materials & methods

### 2.1 Cell culture and treatment

U87, U251, LN229, A172 cell lines were purchased from the cell bank of the Typical Culture Preservation Committee of the Chinese Academy of Sciences (Shanghai, China). The cells were cultured at 37 °C, 5% CO_2_ and 95% air, and kept in DMEM medium supplemented with 10% FBS (Hyclone, USA) and 1% penicillin streptomycin (New Cell & Molecular Biotech., China). The cells were treated with 10 μM SC79 (SF2730, Beyotime Biotech., China) or 5 μM MK2206 (SF2712, Beyotime Biotech., China) for 24 h.

### 2.2 Small interfering RNA (SiRNA) and recombinant plasmid transfection

Small interfering RNA targeting CAV-1 (encoding caveolin-1) and PAI-1 (encoding SERPINE1) were provided by OE Biotech (Shanghai, China). The cells were inoculated into a six-well culture plate and allowed to grow until they reached 60% confluence. As stated in the manufacturer's instructions, Lipofetamine™ 8000 reagent (C0533, Beyotime Biotech., China) was used for genetic modification. After 48 h, we performed functional analysis on the cells, and then detected the transfection rate of the cells by Western blotting.

The pcDNA3.1/caveolin-1 plasmid was transfected into cells using Lipofectamine™8000 reagent. Cells (2 × 10^5^) were seeded per well in antibiotic-free normal growth medium supplemented with 10% FBS until 60% fusion. Lipofectamine™8000 (4 μl) and plasmid (2 μg) were separately incubated with OPTI-MEM (100 μl) at room temperature for 5 min. Subsequently, the two mixtures will be gently mixed together, and the transfection mixture will be added to each well and incubated at 37 °C for 8 h. Finally, the medium was replaced with normal growth medium and the cells were incubated for 24 h, and purinomycin screening was used to obtain stable Cav-1-transfected cells at a concentration of 1000 μg/ml.

### 2.3 Cell counting kit-8 (CCK-8) assay

The CCK-8 assay is used to evaluate cell proliferation ability. Cells transfected with siRNA or plasmids were seeded at a density of 5 × 103 cells per well in a 96-well plate. Incubate the cells with a 1:9 mixture of CCK-8 and basic culture medium for 24, 48, and 72 h. Test the absorbance at 570 nm using a microplate spectrophotometer.

### 2.4 Wound-healing assay

Wound healing assay was conducted to assess the migration ability of golioma cells. Briefly, equal cell quantities were plated in six-well plates and allowed to grow until reaching 100% confluence. Subsequently, wounds were created using a sterile yellow pipette tip, and the cells were washed twice with phosphate-buffered saline (PBS) to eliminate debris. The cells were cultured in DMEM containing 1% FBS at 5% CO2 and 37 °C for 24 h. Wound healing was assessed by comparing the wound area with the initial (0-h) measurement. The experiment was repeated in triplicate and data were presented as the mean ± SD.

### 2.5 Colony formation assay

Cells seeded in six-well plates at a density of 500 cells per well were allowed to grow for 2 weeks. After culturing, the cells were stained with 0.1% crystal violet for 30 min, after fixing with in pre-cooled methanol for 15 min. The plates were washed with PBS. All experiments were repeated three times.

### 2.6 In vitro invasion and migration assays

The transwell inserts (8 μm pore size, NEST, China) were utilized either uncoated or coated with Matrigel, following the manufacturer's instructions. Cells grown in serum-free medium (1.5 × 10^5^ cells/100 μl) were added to the top chamber. 10% FBS was added to the bottom chamber as a chemoattractant. After 48 h of seeding, non-invading cells were removed with a cotton swab, and invading cells were fixed with ice-cold methanol and stained with 0.3% crystal violet. Finally, rinse off excess crystal violet dye with PBS. Images were captured using an inverted microscope (LEICA, Germany). The experiment was repeated in triplicate.

### 2.7 Western blotting

The cells were lysed in RIPA lysis buffer (P0013C, Beyotime Biotech., China) containing proteinase and phosphatase inhibitor cocktails. The protein concentration was determined using a BCA protein assay kit (P0009, Beyotime Biotech., China). Equal amounts of total protein were separated in 8%–15% SDS-polyacrylamide gels and then transferred onto polyvinylidene difluoride membranes. The membranes were blocked using 5% bovine serum albumin, washed, incubated with primary antibodies at 4 °C overnight, washed, and incubated with horseradish peroxidase-conjugated goat anti-rabbit or anti-mouse IgG antibody as appropriate. The protein bands were visualized using enhanced chemiluminescence assay (Thermo Fisher, USA) and exposed to X-ray films. Antibodies for caveolin-1 (GB11409, Servicebio, China), phospho-Akt (T40067S, Abmart, China), Akt (T55561S, Abmart, China), phospho-PI3K(bs-5570R, Bioss, China), PI3K(bs-2067R, Bioss, China), PAI-1(49536S, Cell Signaling Tech., USA), Vimentin (49536S, Cell Signaling Tech., USA), E-Cadherin (14472S, Cell Signaling Tech., USA), N-Cadherin (13116S, Cell Signaling Tech., USA), α-SMA (GB111364, Servicebio, China) were used in western blotting analyses. And β-actin (GB11409, Servicebio, China) was used to confirm equal protein loading.

### 2.8 Quantitative real-time reverse transcription PCR (qRT-PCR)

The TRIzol Reagent (Thermo Fisher Scientific, USA) according to manufacturer's guideline was used to extract total RNA from glioma cells, then approximately 1 mg of total RNA was reverse transcribed via HiScript III RT SuperMix (+gDNA wiper) (Vazyme, Nanjing, China). The quantitative real-time PCR (qRT-PCR) was performed using the AceQ Universal SYBR qPCR Master Mix (Vazyme, Nanjing, China). Relative gene expression levels were calculated using the 2^−ΔΔCt^ method. The primers used are as following: CAV-1, 5′-AGCAAAAGTTGTAGCGCCAG-3′ (forward) and 5′-GACCACGTCGTCGTTGAGAT-3′ (reverse); 18S, 5′-GAACGAGACTCTGGCATGCTA-3′ (forward) and 5′-CACGCTGAGCCAGTCAGTGTA-3′ (reverse); PAI-1, 5′-CTCATCAGCCACTGGAAAGGCA-3′ (forward) and 5′-GACTCGTGAAGTCAGCCTGAAAC-3′ (reverse).

### 2.9 Immunofluorescent staining

The xenograft tissues were fixed overnight with 4% paraformaldehyde and cut into 4 μm thick sections, and permeabilized with 0.1% Triton X-100 for 15 min, Afterwards, the sections were washed three times with PBS. The sections were incubated with the first antibody overnight in a refrigerator at 4 °C. The next day, the first antibody was recovered, the sections were washed three times with PBS, and incubated with an appropriate fluorescent conjugated corresponding secondary antibody at room temperature for 1.5 h. DAPI reagent was used for staining the nucleus. Images were captured using an optical microscope. The expression of α-SMA was analyzed by ImageJ.

### 2.10 H＆E staining and immunohistochemistry staining

The paraffin-embedded Xenograft tissues were sectioned with a thickness of 4 μm. For H&E staining, sections were stained with Haematoxylin and eosin according to standard procedures. For immunohistochemistry staining, the sections were sequentially subjected to xylene dewaxing, ethanol gradient hydration, and citric buffer antigen retrieval. The sections were incubated at room temperature in a humidified chamber with 3% hydrogen peroxide for 10 min. Sections were rinsed with PBS and incubated overnight with primary antibody at 4 °C. The sections were washed and incubated with the corresponding secondary antibody for 30 min the next day, and then DAB and Haematoxylin staining were performed respectively. The sections were incubated at room temperature in a humidified chamber with 3% hydrogen peroxide for 10 min. ImageJ was used for statistics of the positive area of each part.

### 2.11 Xenograft experiments

Animal experiments were strictly conducted in accordance with the principles and procedures approved by the Animal Ethics Committee of the First People's Hospital of Yancheng (Yancheng, China). To evaluate the tumorigenic effect in vivo, 5 × 106 glioma cells with knock down/overexpression of CAV-1 were inoculated into the flanks of 4-week-old male BALB/c nude mice (five mice in each group). The size of the resulting tumor was measured once a week. The tumor volumes were calculated according to the following formula: V = (length × Width 2)/2. After four weeks of inoculation, the mice were euthanized with carbon dioxide and the tumors were dissected and measured. The data were presented as Mean ± SD.

### 2.12 Data acquisition and preprocessing

RNA-seq data in TPM format were collected from the Cancer Genome Atlas (TCGA) database and the Genotype Tissue Expression Project (GTEx) database available at UCSC Xena (https://xenabrowser.net/datapages/), these data were processed uniformly through the tour process. The glioma data corresponding to TCGA and the normal tissue data corresponding to GTEx were obtained. The R-package GOplot (version 1.0.2) was used to visualize the data.

### 2.13 Differentially expressed gene analysis

According to the median expression level of CAV-1, the expression data (HTseq count) were divided into high expression and low expression groups, and further analysis was conducted using unpaired Student’s t-test within the DESeq2 R package (3.6.3). The adjusted p < 0.05 and | log 2-fold change (FC) |>1.5 were considered as the thresholds for DEGs.

### 2.14 Enrichment analysis

The R package GOplot (version 1.0.2) was used for functional enrichment analysis of DEGs between high and low expression levels of CAV-1, including Gene Ontology (GO) and Kyoto Encyclopedia of Genes and Genomes (KEGG) analysis. The use of ClusterProfiler software package in R (3.6.3) for gene set enrichment analysis (GSEA), with an adjusted p-value of <0.05 and an error detection rate (FDR) of <0.025, is considered a statistically significant enrichment of functional or pathway items.

### 2.15 Immune infiltration analysis

Based on the single-sample GSEA algorithm provided in the R package GSVA, the immune infiltration status of the corresponding data was calculated using the signature genes of 24 immune cells provided in the published article [20]. The infiltration of immunocytes between the CAV-1 high and low expression group was analyzed by Spearman's correlation analysis and the Wilcoxon rank-sum test.

### 2.16 Statistical analysis

All experiments for cell cultures were repeated independently for at least three times and in triplicate each time. GraphPad Prism 5.0 was used for analyzation. A two-tailed unpaired *t*-test and ANOVA were used to determine significant differences. A p-value <0.05 was considered significant.

## 3 Results

### 3.1 CAV-1 is upregulated in glioma

To investigate the underlying role of CAV-1 in glioma, we utilized TCGA and GTEx database to acquire RNA-Seq data from 689 glioma patients and 1152 normal brain tissues. The unpaired differential expression analyses between normal and glioma groups revealed significantly higher expression of CAV-1 in tumors compared to normal tissue (Fig. 1A). The ROC analysis of CAV-1 supported the diagnostic accuracy of the score (AUC = 0.855, 95% CI: 0.838–0.872) (Fig. 1B). Moreover, IHC results also demonstrated higher CAV-1 expression in glioma tissues compared to the corresponding adjacent normal tissues (Fig. 1C). Next, we detected the expression of CAV-1 in glioma cell lines (U87, U251, LN229, A172). The results showed that in glioma cell lines, the expression of CAV-1 in U87 was high, and the expression of CAV-1 in U251 cells was low (Fig. 1D and E). Therefore, we selected U87 and U251 cell lines for subsequent experimental research. In conclusion, these findings suggest that CAV-1 is upregulated in glioma.

**Fig. 1:**
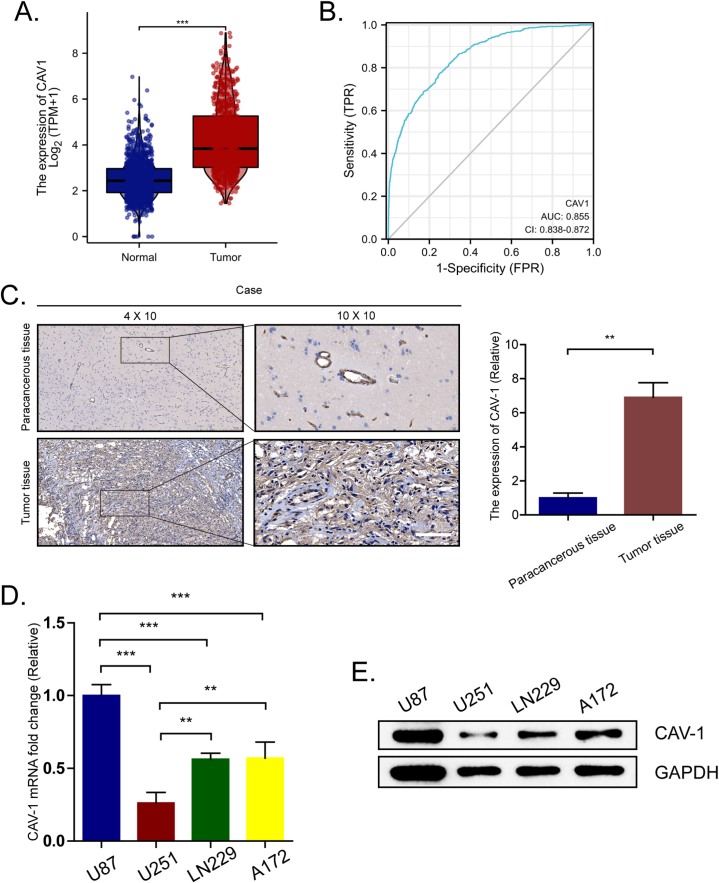
CAV-1 is upregulated in glioma, **(A)** The different expressions of CAV-1 between glioma and the normal group in the TCGA and GTEx databases. **(B)** ROC curves for classifying glioma versus the normal group in the TCGA database. **(C)** Representative images of CAV-1 expression in glioma and their matched paracancerous tissues. **(D**–**E)** Relative mRNA and protein expression of CAV-1 in four glioma cell lines.

### 3.2 Upregulation of CAV-1 promotes glioma cell proliferation and metastasis in vitro

To investigate the biological functions of CAV-1 in glioma cells, CAV-1 was overexpressed in U251 cells, while CAV-1 was knocked down in U87 cells (Fig. 2A). Notably, the silencing of CAV-1 in U87 cells significantly reduced the proliferative capacity of glioma cells, as evaluated by CCK-8 and colony formation assays (Fig. 2C and D). Moreover, according to Transwell and wound-healing assays (Fig. 2B–E), CAV-1 silencing decreased glioma cells invasion and migration.

**Fig. 2:**
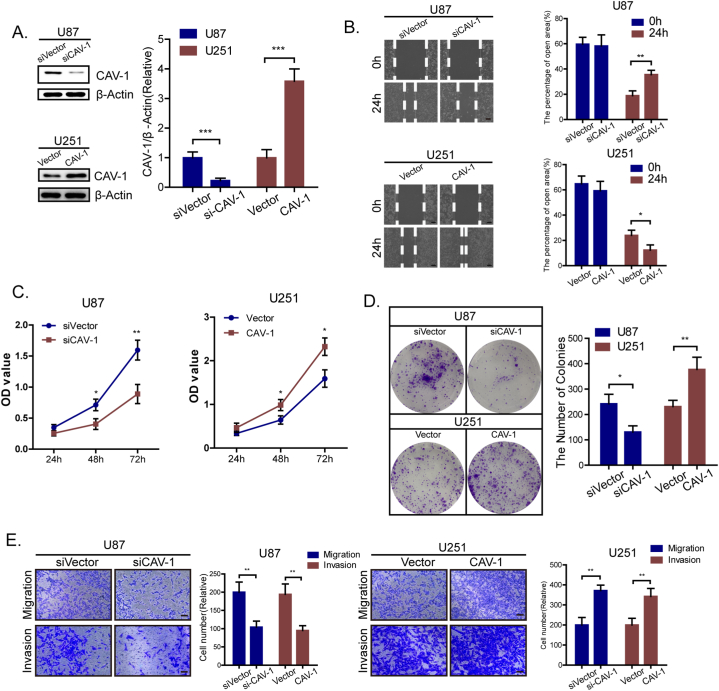
Upregulation of CAV-1 promotes glioma cell proliferation and metastasis in vitro **(A)** Western blot analysis confirms the successful knockdown of CAV-1 in U87 cells and the effective overexpression of CAV-1 in U251 cells. **(B)** Presented are representative data from wound healing assays conducted using the specified glioma cells. Scale bar = 100 μm. **(C**–**D)** Representative data from Cell counting kit-8 assay and Colony formation assay performed with the indicated glioma cells. **(E)** Representative data from Transwell migration and Matrigel invasion assays performed with the indicated glioma cells. The data are the means ± SD and are representative of three independent experiments; *, *p < 0.05*; **, *p < 0.01*; ***, *p < 0.001*; *ns, not significant.*

By contrast, overexpression of glioma in U251 cells increased the proliferative capacity significantly. Importantly, Transwell assays showed that overexpression of CAV-1 enhanced the invasion and migration of U251 cells. Similarly, in a wound-healing assay, overexpression of CAV-1 led to increased migration potential. Therefore, these findings suggest that CAV-1 can promote the proliferation and metastasis of glioma cells in vitro.

### 3.3 CAV-1 promotes the EMT process in glioma cells through activating PI3K/Akt signaling pathway

Functional annotations were performed using GO and KEGG analyses with CAV-1. The functional annotations demonstrated that DEG-related CAV-1 had significant regulation on signaling receptor activator activity, receptor ligand activity, collagen-containing extracellular matrix (Fig. 3A). The KEGG molecular pathways were neuroactive ligand-receptor interaction, cytokine-cytokine receptor interaction and PI3K-Akt signaling pathway (Fig. 3B). To further identify the biological function of CAV-1, the GSEA of differences between low and high CAV-1 expression data sets were performed to identify the Hallmark associated with CAV-1. As shown in Fig. 3C, epithelial mesenchymal transition is the most differentially enriched pathway in CAV-1 high expression phenotype, suggesting that the high expression of CAV-1 might conferred EMT process in glioma.

**Fig. 3:**
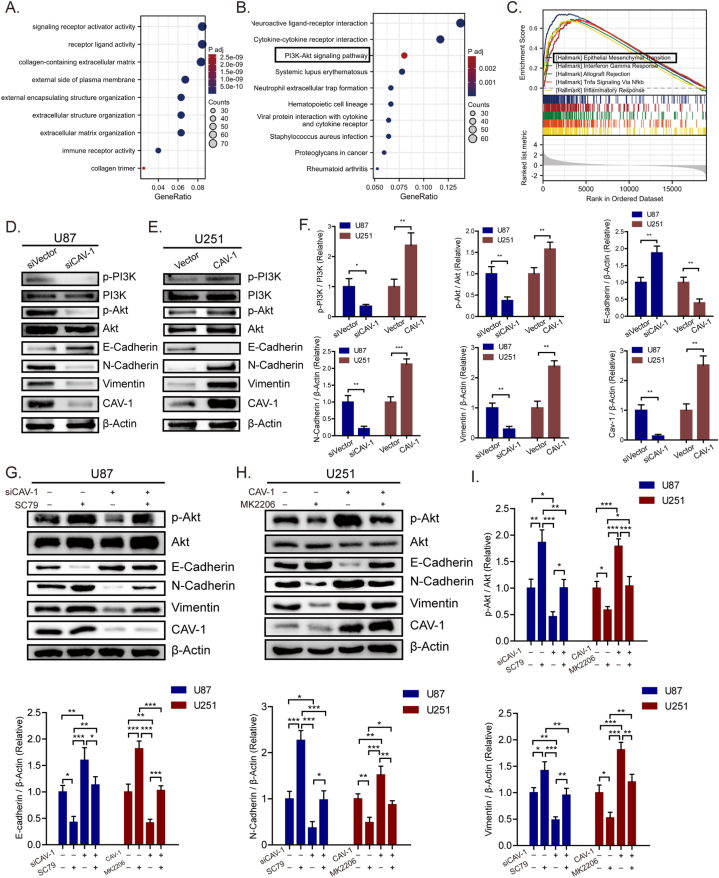
CAV-1 promotes the EMT process in glioma cells through activating PI3K/Akt signaling pathway **(A**–**B)** Enrichment analysis of biological processes associated with CAV-1-related genes. **(C)** GSEA analysis of the Hallmark gene sets deposited in MSigDB. **(D**–**E)** Relative expression levels of CAV-1, PI3K, Akt, E-cadherin, N-cadherin and Vimentin in the indicated cells. **(F)** Quantified protein expression levels of CAV-1, p-PI3K/PI3K, p-Akt/Akt, E-cadherin, N-cadherin and Vimentin. **(G**–**H)** Relative expression levels of CAV-1, PI3K, Akt, E-cadherin, N-cadherin and Vimentin in the indicated cells treated with SC79 or MK2206. **(I)** Quantified protein expression levels of CAV-1, p-Akt/Akt, E-cadherin, N-cadherin and Vimentin. The experiment was repeated in triplicate and results are presented as mean ± SD; *, *p < 0.05*; **, *p < 0.01*; ***, *p < 0.001*; *ns, not significant.*

The phosphatidylinositol 3-kinase/protein kinase B (PI3K/Akt) signaling pathway is widely activated in human cancer and plays a crucial role in promoting various tumor cell processes such as survival, proliferation, metabolism, invasion, and angiogenesis [21]. Based on the above analysis results, we speculated that CAV-1 regulates the EMT process of glioma cells through the PI3K/Akt signaling pathway. In this study, we first evaluated the changes in the expression of this signaling protein. Western blotting showed that compared to control, CAV-1 expression was significantly downregulated or upregulated in CAV-1-siRNA or CAV-1 over-expressed cells. Subsequent downregulation of CAV-1 decreased the levels of p-PI3K, p-AKT, N-cadherin, and Vimentin proteins in glioma cells, while increasing E-cadherin proteins, without significant effects on the total levels of PI3K and AKT (Fig. 3D–F). Over-expression of CAV-1 induced the expression of p-PI3K, p-AKT, N-cadherin and Vimentin proteins in glioma cells, while decreasing E-cadherin proteins, without significant effects on the total levels of PI3K and AKT (Fig. 3E and F). Subsequently, we performed Western blotting analysis to confirm the association between CAV-1 promotion of EMT in glioma cells and the PI3K/AKT signaling pathway. The results showed that CAV-1 depletion in U87 cells decreased the expression levels of mesenchymal markers N-cadherin and increased the expression levels of endothelial marker E-cadherin, while these effects were halted following the addition of 10 μM SC79(Akt agonist) (Fig. 3G–I). However, after adding 5 μM MK2206 (Akt inhibitor) to U251 cells with CAV-1 over-expression, the expression levels of N-cadherin and Vimentin decreased, and the expression level of E-cadherin increased (Fig. 3H and I), indicating that activation of the Akt signaling pathway reversed the inhibitory effect of CAV-1 knockdown on EMT. These results suggested that CAV-1 promotes EMT in glioma cells by activating the PI3K/Akt signaling pathway.

### 3.4 The role of PAI-1 in CAV-1-induced activation of PI3K/Akt signaling and EMT in glioma cells

To further investigate the molecular mechanism of CAV-1-mediated PI3K/Akt signaling upregulation. We conducted a series of bioinformatics analyses. The correlation between the top 25 genes and CAV-1 were presented in a co-expression heat map (Fig. 4A). With |logFC| > 1.5 and adjusted p < 0.05 set as the cut-off criteria, a total of 919 DEGs were identified by analyzing the HTSeq-Counts data of CAV-1-related genes from TCGA. 919 DEGs of CAV-1, 1030 significantly co-expressed genes and 128 EMT gene were identified by Draw Venn diagrams online tool, 48 overlapping genes were identified for further analysis (Fig. 4B). To explore the expression correlation of these 48 EMT-related genes, correlation analysis was performed. Among these 48 genes, SERPINE1 (PAI-1) exhibited the strongest co-expression correlation with CAV-1 (Fig. 4C). Furthermore, among the identified 919 DEGs, SERPINE1 (PAI-1) demonstrated the most significant expression differences (Fig. 4D). Moreover, there is a significant correlation between SERPINE1 and PI3K genes (R = 0.555, P < 0.001) (Fig. 4E and F). Down-regulated the expression of CAV-1 reduced the expression of PAI-1 in U87 cells, while over-expressed CAV-1 increased the expression of PAI-1 in U251 cells (Fig. 4G), suggesting that PAI-1 may be involved in the PI3K/Akt signaling process regulated by CAV-1.

**Fig. 4:**
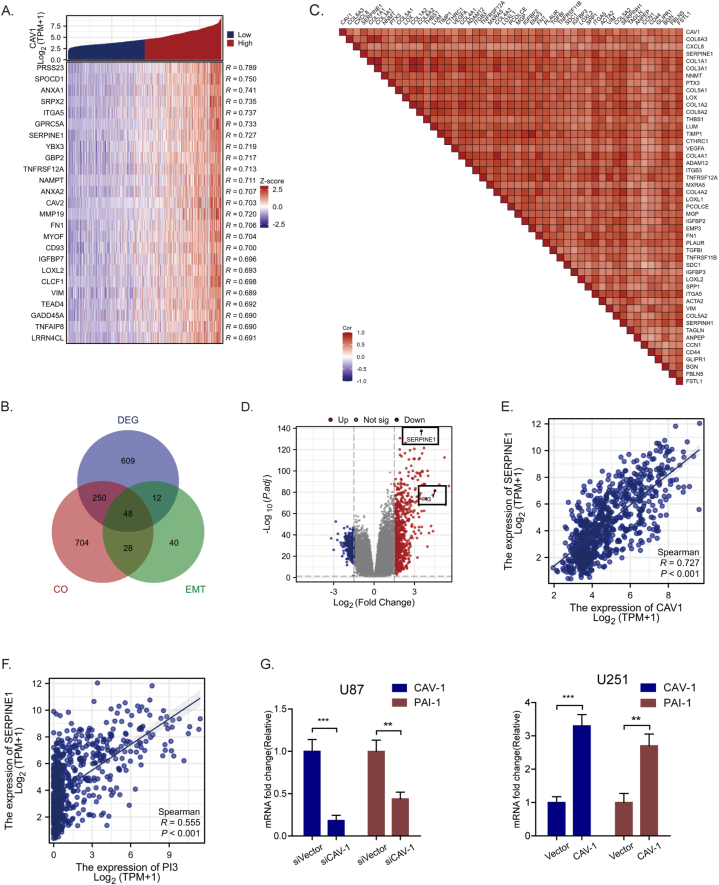
PAI-1 appears to play a role in the PI3K/Akt signaling process regulated by CAV-1. **(A)** The heat map of the 25 genes correlated to CAV-1. **(B)** Venn diagram of the overlapping genes among significantly co-expressed genes, significantly DEGs and EMT-associated genes of CAV-1. **(C)** Spearman correlation between CAV-1 and 48 Overlapping genes. **(D)** Volcano plot of DEGs. Blue and red dots indicate the significantly down-regulated and up-regulated DEGs, respectively. DEGs, differentially expressed genes. **(E)** The positive correlation between CAV-1 expression and SERPINE1(PAI-1) expression. **(F)** The positive correlation between PI3K expression and SERPINE1(PAI-1) expression.

### 3.5 PAI-1 plays a vital role in the CAV-1-induced PI3K/Akt signaling activation and EMT in glioma cells

To verify whether CAV-1 activated PI3K/Akt signaling in glioma cells through regulation of PAI-1, we detected the levels of PI3K and Akt in PAI-1-silenced cells with CAV-1 over-expression and observed that PAI-1 knockdown decreased the expression of p-PI3K, p-Akt, Vimentin and N-cadherin, and the expression of these proteins were not reversed following the over-expression of CAV-1 (Fig. 5A and B). Functional analysis confirmed these results, as knockdown of PAI-1 abolished the EMT-mediated enhancement of cell proliferation, migration, and invasion. Importantly, these inhibitory effects were not restored upon CAV-1 overexpression (Fig. 5C–F). These results suggested that PAI-1 was crucial for the CAV-1-induced PI3K/Akt signaling activation and EMT in glioma cells.

**Fig. 5:**
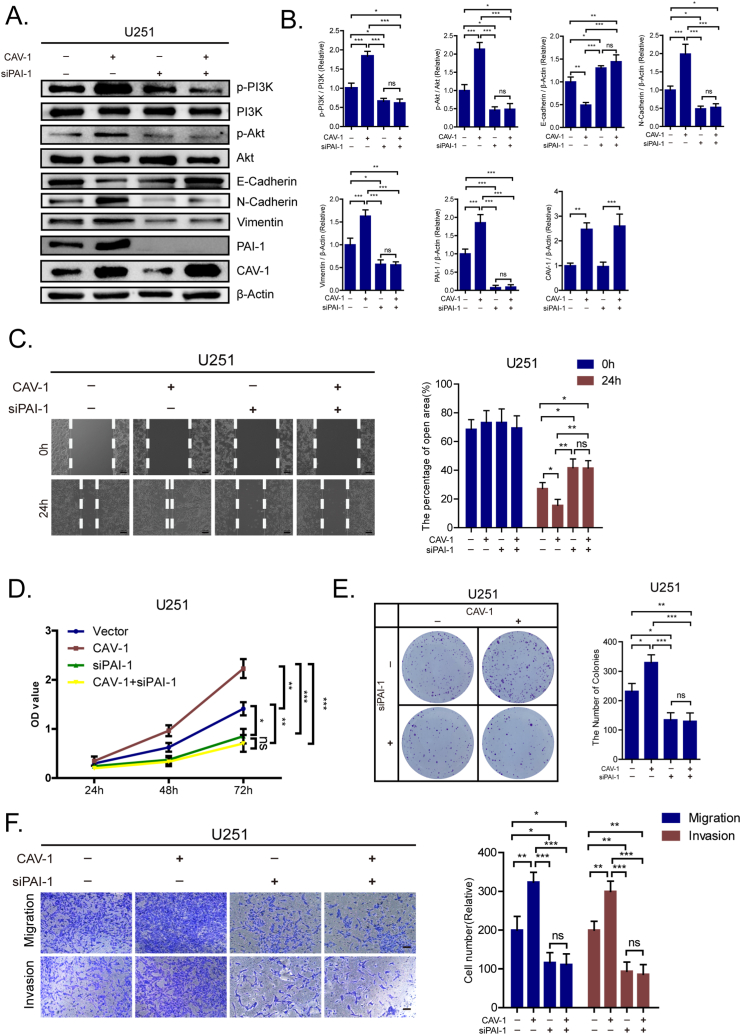
The Role of PAI-1 in CAV-1-induced activation of PI3K/Akt signaling and EMT in glioma Cells **(A)** Western blotting analysis of the levels of p-PI3K/PI3K, p-Akt/Akt, Vimentin, N-cadherin, and E-cadherin in U251 cells co-transfected with CAV-1 or PAI-1 siRNAs. **(B)** Quantified protein expression levels of p-PI3K/PI3K, p-Akt/Akt, Vimentin, N-cadherin, and E-cadherin. **(C)** Wound healing assays and performed with the indicated U251 cells co-transfected with CAV-1 or PAI-1 siRNAs. **(D**–**E)** Representative data from Cell counting kit-8 assay and Colony formation assay performed with the U251 cells co-transfected with CAV-1 or PAI-1 siRNAs. **(F)** Representative data from Transwell migration and Matrigel invasion assays performed with the U251 cells co-transfected with CAV-1 or PAI-1 siRNAs.

### 3.6 Knockdown of CAV-1 inhibits glioma growth and angiogenesis in vivo

To investigate the in vivo effect of CAV-1 on tumor growth, CAV-1-transduced cells and control U251/U87 cells were injected subcutaneously into nude mice’s right shoulders. Four weeks later, the size and weight of the tumour in the CAV-1 knockdown group was considerably decreased compared with that in the control U87 group, the size and weight of the tumour in the CAV-1 over-expression group was considerably increased compared with that in the control U251 group (Fig. 6A–D). IHC was performed on the Xenograft tissues, revealing that CAV-1 knockdown led to a decrease in N-Cadherin and Vimentin expression and an increase in E-Cadherin expression. Conversely, CAV-1 overexpression resulted in an increase in N-Cadherin and Vimentin expression and a decrease in E-Cadherin expression. Furthermore, we observed a significant decrease in the expression of Ki-67 in the CAV-1 knockdown group, whereas the CAV-1 overexpression group exhibited an increase in Ki-67 expression (Fig. 6E). These results indicated that knockdown of CAV-1 inhibits the growth, EMT of glioma in vivo, and ultimately suppressed the tumorigenicity of glioma.

**Fig. 6:**
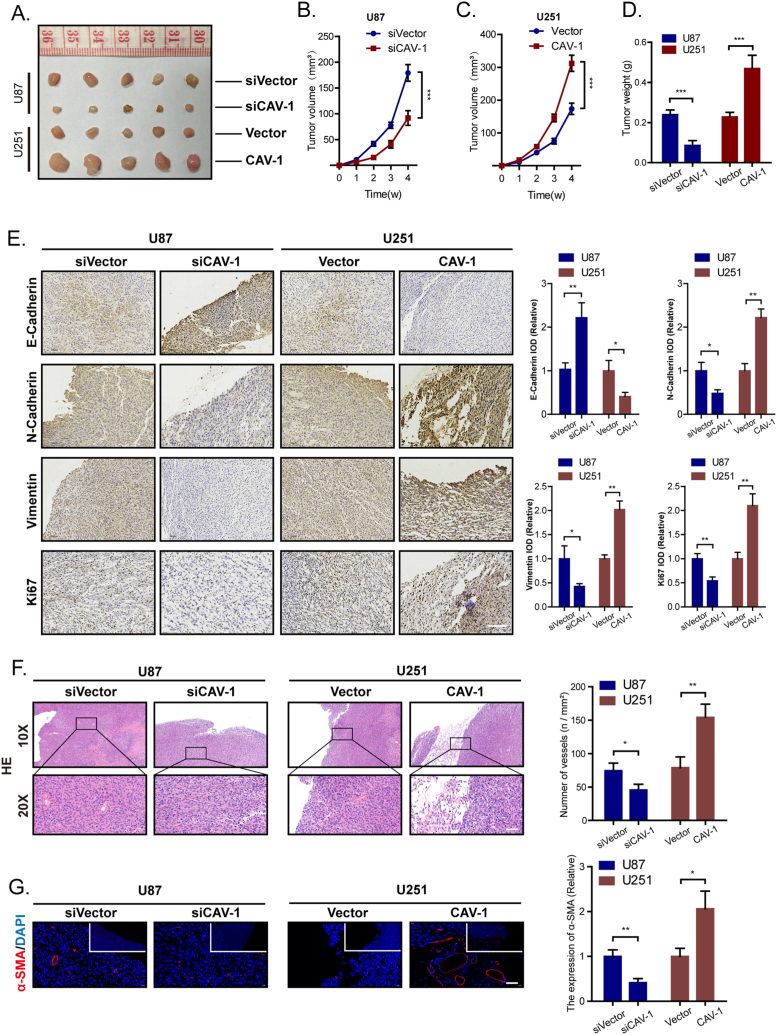
Knockdown of CAV-1 inhibits glioma growth and angiogenesis in vivo. Glioma cells with either suppressed or overexpressed CAV-1 were introduced into the flanks of BALB/c nude mice. After four weeks of inoculation, the tumors were dissected and measured. (A) Glioma images (scale bar 1 cm). (B–C) Statistical analysis of tumor volume after tumors separated. **(D)** Statistical analysis of tumor weight. **(E)** Representative immunohistochemical staining of E-Cadherin, N-Cadherin, Vimentin, and Ki67 in glioma tissues; scale bar: 100 μm. **(F)** The Relative IOD in glioma tissues of E-Cadherin, N-Cadherin, Vimentin, and Ki67. **(G)** H＆E staining used to detect the angiogenesis on glioma tumors (scale bar 200 μm) and quantified the number of vessels. **(H)** Immunofluorescence used to detect the expression of α-SMA on glioma tumors (scale bar 200 μm) and quantified the expression of α-SMA.

Angiogenesis, the process that facilitates the growth of new capillary blood vessels, is a crucial process in the growth, maintenance and metastasis of solid tumors [22]. Angiogenesis is a highly complex process involving endothelial cell proliferation, migration, invasion and tube formation. Mounting evidence suggests that glioma development and metastasis critically depend on angiogenesis, and the levels of tumor angiogenesis are associated with the prognosis of glioma patients [23,24]. Recognizing the importance of angiogenesis in tumor progression, some scholars proposed the concept of ‘anti-angiogenic therapy’ [[25], [26], [27]]. In this study, we observed a significant decrease in angiogenesis in the CAV-1 silenced U87 group compared to the control groups. Additionally, angiogenesis was significantly increased in the U251 group following the over-expression of CAV-1, as demonstrated by H&E staining (Fig. 6F). α-SMA, a vascular smooth muscle marker, was used to evaluate the degree of angiogenesis. Consistent with the data of H&E staining, the expression of α-SMA decreased significantly in the CAV-1 silenced U87 group, while its expression was significantly increased in the U251 group after over-expression of CAV-1 (Fig. 6G). Our data demonstrated that knockdown of CAV-1 inhibits glioma angiogenesis.

### 3.7 Role of CAV-1 in immune infiltration

Spearman correlation was used to investigate the correlation between the CAV-1 expression level in the form of TPM and the immune cell infiltration level quantified as the ssGSEA score. The results showed that the level of cell infiltration of macrophage cells (Spearman R = 0.637, *p < 0.001*), neutrophils (Spearman R = 0.574, *p < 0.001*) and immature dendritic cells (iDCs) (Spearman R = 0.470, *p < 0.001*) were significantly positively correlated with the expression of CAV-1 (Fig. 7A, D–F). And the enrichment scores of macrophage cells, neutrophils and immature dendritic cells in the CAV-1 high expression group were higher than those in the CAV-1 low expression group (*p < 0.001*) (Fig. 7B). The degree of infiltration of plasmacytoid dendritic cells (pDCs) (Spearman R = −0.332, *p < 0.001*), NK CD56bright cells (Spearman R = −0.280, *p < 0.001*) and Follicular helper T cell (TFH) (Spearman R = −0.230, *p < 0.001*) were negatively correlated with CAV-1 expression (Fig. 7A, G–I) and were significantly lower in the CAV-1 high expression group (p < 0.001) (Fig. 7C). These results suggested that CAV-1 plays an important role in the immune infiltration of glioma.

**Fig. 7:**
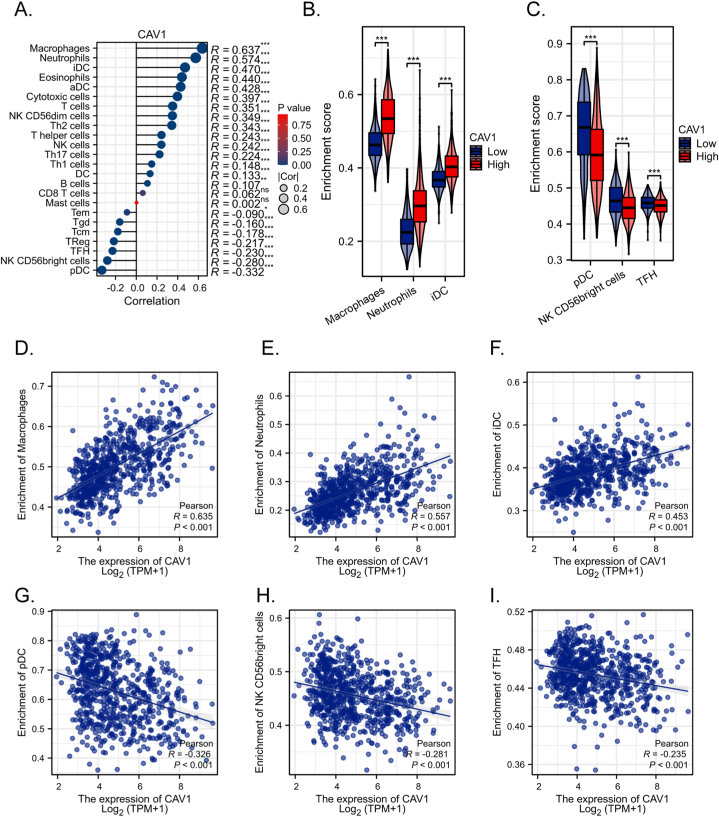
**(A)** Correlation between CAV-1 expression and the relative abundances of 24 types of immune cell. **(B–C)** Comparison of immune infiltration levels of immune cells between the different CAV-1 expression groups. **(D**–**I)** Correlations between the relative enrichment scores of immune cells and the expression of CAV-1. *, *p < 0.05*; **, *p < 0.01*; ***, *p < 0.001*; *ns, not significant.*

### 3.8 Upregulation of CAV-1 correlates with the dismal prognosis in glioma patients

To clarify the underlying role of CAV-1 in glioma, we first analyzed the associations between CAV-1 expression and clinicopathologic variables. Welch one-way ANOVA followed by the Bonferroni correction proved that the expression of CAV-1 was significantly correlated with the WHO grade and pathologic stage (Fig. 8A and B). The *t*-test revealed that the expression of CAV-1 was significantly correlated with the IDH status, 1p/19q codeletion, OS event and DSS event (Fig. 8C–F). The Kaplan–Meier analysis for CAV-1 revealed that patients with higher CAV-1 levels had a shorter post-operative OS and DSS (Fig. 8G and H). According to time-dependent ROC, the CAV-1 expression level had a relatively good performance in predicting 1-year, 3-year, 5-year OS and DSS in glioma patients (Fig. 8I and J). Multivariate Cox regression showed that CAV-1 (*p < 0.001*) was independent prognostic factor for OS (Fig. 8K). The distribution of CAV-1 expression, survival status of glioma patients, and expression profiles of CAV-1 and PAI-1 are shown in Fig. 8L, the left side of the upper line represents the low-risk score group with low CAV-1 expression, and the right side of the dotted line represents the high-risk score group with high CAV-1 expression. As the risk score of glioma patients increases, the number of red dots increased, and the number of dead glioma patients increased. It showed that high-risk group have a lower survival rate and a higher risk of death. In summary, these findings suggest a correlation between upregulation of CAV-1 and poor prognosis in patients with glioma (see Fig. 9).

**Fig. 8:**
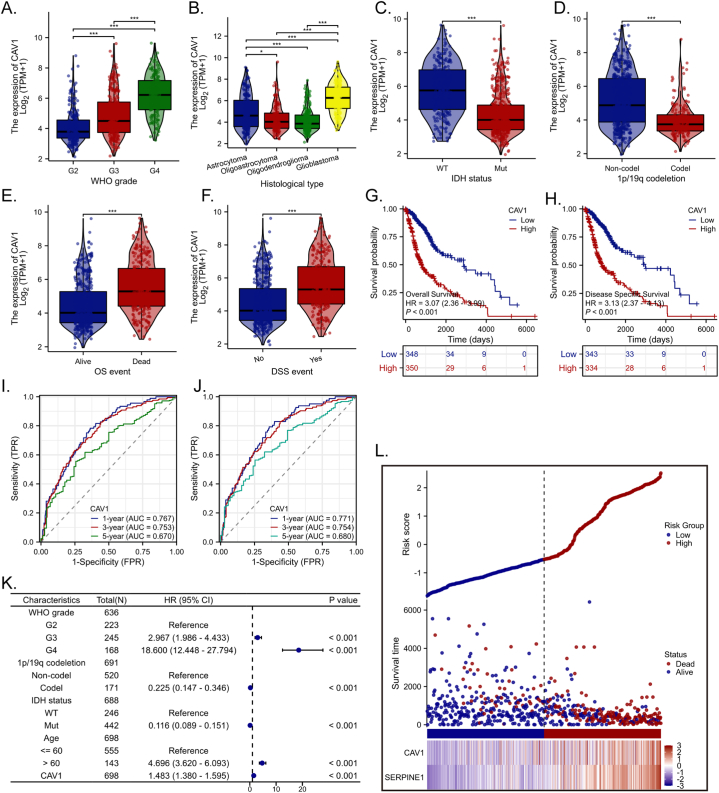
Upregulation of CAV-1 correlates with the dismal prognosis in glioma patients The expression of CAV-1 in different grade **(A)** histological type **(B)** IDH status **(C)**, 1p/19q **(D)**, OS **(E)**, DSS **(F)**, Overall survival **(G)** and disease-specific survival **(H)** for glioma patients with high versus low CAV-1. The predictive efficiency of the OS and DSS were verified by the ROC curve **(I, J)**. **(K)** Multivariate Cox regression visualized in the forest plot. **(L)** CAV-1 expression distribution and survival status.

**Fig. 9:**
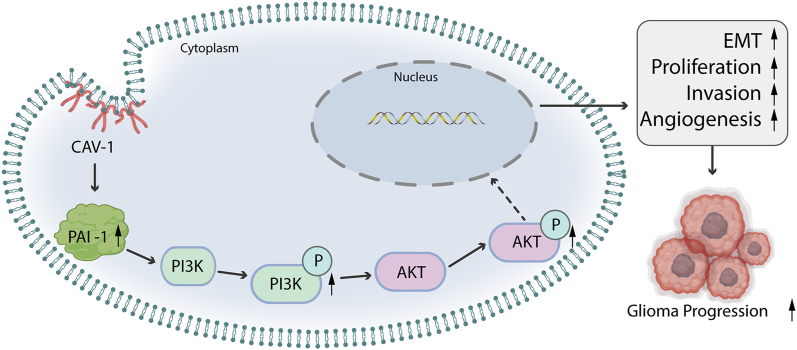
Schematic diagram of the possible mechanisms of action of CAV-1 on glioma.

## 4 Discussion

Metastasis poses a significant challenge in tumor treatment. For glioma patients with distant metastasis, there is very little opportunity for surgery. Temozolomide is the most commonly used chemotherapeutic drug for glioma [28,29]. Nevertheless, Temozolomide is associated with adverse effects such as dizziness, nausea, leukopenia, thrombocytopenia, aplastic anemia, and B-cell non-Hodgkin's lymphoma [30,31]. This study reveals CAV-1 as a novel regulator of SERPINE1. As a partial epithelial mesenchymal transition marker, SERPINE1 plays a crucial role in tumor proliferation and metastasis, identification of the upstream factors of SERPINE1 may provide potential therapeutic targets. However, the potential of CAV-1 as a drug target requires further evaluation.

Recent studies have demonstrated the significant involvement of CAV-1 in cancer development and progression [32]. CAV-1 exhibits high expression in a variety of malignant tumors and exerts a crucial influence on tumor metastasis [33,34]. Nevertheless, the role of CAV-1 in glioma remains elusive. This study revealed that CAV-1 stimulates the in vitro proliferation, migration, and invasion of glioma cells, while also promoting the growth and angiogenesis of glioma cells in a mouse xenograft tumor model. More importantly, we elucidated the molecular mechanism by which CAV-1 regulates glioma metastasis. Additionally, our analysis revealed a correlation between upregulated CAV-1 expression in glioma patients and an unfavorable prognosis. As the WHO grading increased, the expression of CAV-1 also increased. CAV-1 expression increased in the IDH-WT and 1p/19q non-codel, and previous studies have shown that patients with IDH-WT or 1p/19q non-codel have poor prognosis [35,36]. These results suggested that upregulation of CAV-1 is associated with poor prognosis in glioma patients. Our findings contribute to the comprehension of CAV-1's function and facilitate the assessment of its potential as a biomarker or therapeutic target for glioma. Cancer cell migration and invasion are dependent on EMT and angiogenesis [37,38]. EMT involves down-regulation of epithelial markers (E-cadherin, ZO-1, and Occludin) and up-regulation of mesenchymal markers (Vimentin and N-cadherin) [39]. A previous study reported that CAV-1 is involved in EMT regulation in non-small cell lung cancer and hepatocellular carcinoma [40,41]. However, its association with EMT in glioma cells remains unclear. In this study, we observed that CAV-1 knockdown increased the expression of epithelial marker E-cadherin and decreased the expression of mesenchymal markers Vimentin and N-cadherin. Conversely, CAV-1 overexpression reduced the expression of E-cadherin and increased the expression of Vimentin and N-cadherin. Therefore, these results indicated that CAV-1 promotes EMT in glioma cells.

Angiogenesis is another important factor to promote cancer cells migration and invasion [42]. Angiogenesis refers to the formation of new blood vessels from the preexisting vascular system, enabling the transportation of oxygen and nutrients to the tumor, thereby facilitating its progression [43,44]. Consequently, angiogenesis has been acknowledged as a significant hallmark of cancer. During the occurrence and development of tumor, the balance between pro-angiogenic factors and anti-angiogenic factors is disrupted, leading to the activation of the ‘angiogenesis switch’ [45,46]. Previous studies have shown that cytokines secreted from cells in the tumor microenvironment can mediate the influence on different cells in tumor microenvironment and activate the signal pathway involved in angiogenesis [47]. In this study, we found that knockdown of CAV-1 inhibited angiogenesis in glioma and reduced the expression of α-SMA, a vascular smooth muscle marker commonly used to evaluate angiogenesis. Conversely, over-expression of CAV-1 had a promoting effect on angiogenesis, and α-SMA expression was enhanced. These results indicated that CAV-1 promotes angiogenesis in glioma.

The PI3K/AKT signaling pathway is widely recognized as a crucial pathway involved in various normal cellular processes [48]. Abnormal activation of this pathway in numerous human cancers regulates important cellular processes such as autophagy, EMT, apoptosis, chemotherapy resistance, and metastasis [49]. Importantly, the PI3K-Akt signaling pathway is a potential target for multiple genes to promote the proliferation and migration of various types of cancer cells [50,51]. Previous studies have demonstrated a close relationship between the PI3K/Akt signaling pathway and EMT in tumors. Xu et al. identified that FAT4 plays a partial regulatory role in EMT and autophagy within colorectal cancer cells via the PI3K/AKT signaling axis [52]. Wang et al. found that TEAD4 functions as a prognostic biomarker and triggers EMT via PI3K/AKT pathway in bladder cancer [53]. In this experiment, we discovered that CAV-1 activates the PI3K/Akt signaling pathway, triggering the process of EMT and promoting glioma progression.

SERPINE1, also known as PAI-1, serves as the main inhibitor of plasmin activator, which is related to the occurrence and development of a variety of tumors, including breast cancer, glioma, colon cancer, etc [54]. PAI-1 regulates tumor growth by promoting angiogenesis and participates in the migration and invasion of cancer cells [55,56]. It is reported that PAI-1 is also related to multiple drug resistance [57]. Therefore, PAI-1 is also considered as a partial marker for EMT [58]. Previous studies have reported the connection between PAI-1 and the PI3K/AKT signaling pathway. For example, the combination of PAI-1 inhibitors and cisplatin exerts a synergistic effect by inhibiting the PI3K/AKT pathway in glioma. Another study revealed that PAI-1 activated PI3K/Akt signaling pathway and promoted the metastasis of triple negative breast cancer cells [59,60]. In this experiment, to further demonstrate whether PAI-1 plays an important role in the regulation of PI3K-AKT pathway by CAV-1, we measured the levels of Akt and PI3K in PAI-1-silenced cells. Our findings indicated a decrease in p-Akt and p-PI3K, while Akt and PI3K did not exhibit significant changes. However, overexpression of CAV-1 in PAI-1-silenced cells did not lead to an increase in the expression of p-Akt and p-PI3K. Functional experiments indicated that the absence of PAI-1 inhibits glioma cells proliferation, migration, and invasion, indicating that PAI-1 plays an important role in promoting glioma metastasis.

Numerous studies have shown that the immune system plays an important role in the occurrence and development of tumors, and the infiltration characteristics of immune cells are related to the efficacy and clinical efficacy of immunotherapy [61]. Although immunotherapy has achieved significant efficacy in most types of cancer, the effectiveness of immunotherapy drugs in gliomas is greatly reduced due to the presence of the blood-brain barrier (BBB) [62]. Therefore, studying the immunological status has a profound impact on the treatment strategies for glioma. In our study, we calculated the correlation between the expression of CAV-1 and the level of immune infiltrating cells, and the results showed a positive correlation between the expression of CAV-1 and infiltration of macrophages, neutrophils, iDCs, etc. According to previous research, activating the polarization of tumor-associated macrophages reshapes the tumor microenvironment and promotes tumor development [63]. Neutrophils are known as important contributors to tumor progression and metastasis, promoting their progression in various tumors [64]. Ofer Feinaru et al. found that the presence of immature dendritic cells was directly related to the expression of angiogenic phenotype through the study of treating breast cancer and glioblastoma. In fast-growing “angiogenic” tumors, compared with their respective dormant avascular tumors, they were infiltrated by more immature DC populations, and immature DCs promoted tumor growth. Therefore, the mature state of dendritic cells determines their direct proangiogenic and cancer promoting properties [65]. Xu et al.'s study also found that the infiltration of mature DCs in tumors is associated with long-term survival, while the presence of imDCs within tumors may promote tumor progression and lead to poor prognosis [66]. On the other hand, we found a negative correlation between the expression of CAV-1 and infiltration of plasma like pDC, NK cells, and TFH, etc. As is well known, pDC and NK cells promote anti-tumor immunity in the tumor microenvironment [67,68]. TFH cells set the stage for tumor control [69]. These results indicate that the expression of CAV-1 may affect the progression and prognosis of glioma by regulating the level of infiltrating immune cells.

## 5 Conclusions

In summary, our study demonstrated that CAV-1 upregulates the PI3K/Akt signaling pathway, which is activated by PAI-1 (Fig. 8). Additionally, CAV-1 regulates the epithelial-mesenchymal transition (EMT) and angiogenesis in glioma cells, thus promoting glioma metastasis. Overexpression of CAV-1 in glioma is a strong indicator of tumor high invasiveness and is associated with poor clinical prognosis and immune infiltration. Studying the biological function and potential molecular mechanisms of CAV-1 in glioma could help us understand the proliferation and metastasis mechanisms of glioma. In conclusion, our study identifies CAV-1 as a crucial player in glioma proliferation and metastasis and immune cell infiltration. Moreover, it has the potential to serve as a prognostic biomarker and therapeutic target for glioma.

## Data availability statement

The original contributions presented in the study are included in the article. All the raw data have been deposited in Figshare as per your request. Below are links to raw data by Figshare: https://figshare.com/s/075ffca3c0d58c21e4ed.

## Ethics approval

Experiments involving the use of animals were reviewed and approved by the Animal Ethics Committee of the First People's Hospital of Yancheng with approval number No. IACUC20231215-1001.All methods were carried out according to the guidelines and regulations for animal experimentation, and all methods are reported in compliance with the ARRIVE guidelines (https://arriveguidelines.org) for the reporting of animal experiments.

## CRediT authorship contribution statement

**Zhaoxiang Wang:** Writing – original draft, Methodology, Investigation, Formal analysis. **Gang Chen:** Writing – original draft, Methodology, Investigation, Formal analysis. **Debin Yuan:** Validation, Conceptualization. **Peizhang Wu:** Validation, Conceptualization. **Jun Guo:** Validation, Conceptualization. **Yisheng Lu:** Validation, Conceptualization. **Zhenyu Wang:** Writing – review & editing, Methodology, Investigation, Formal analysis.

## Declaration of competing interest

The authors declare that they have no known competing financial interests or personal relationships that could have appeared to influence the work reported in this paper.

## Figures and Tables

**figs1:**
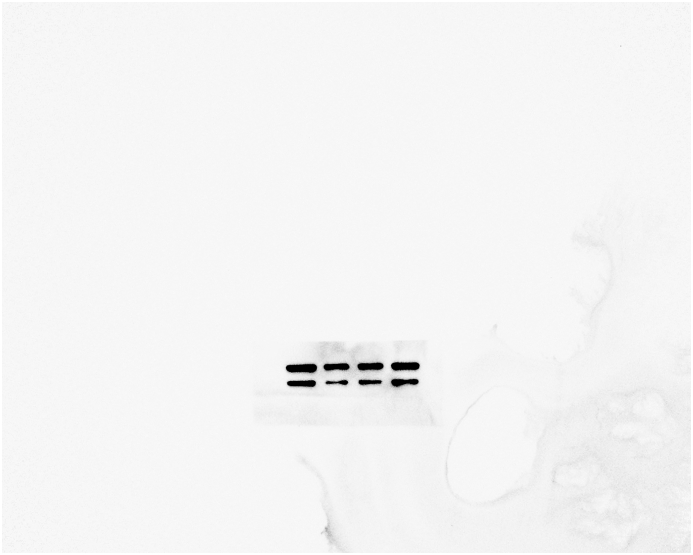


**figs2:**
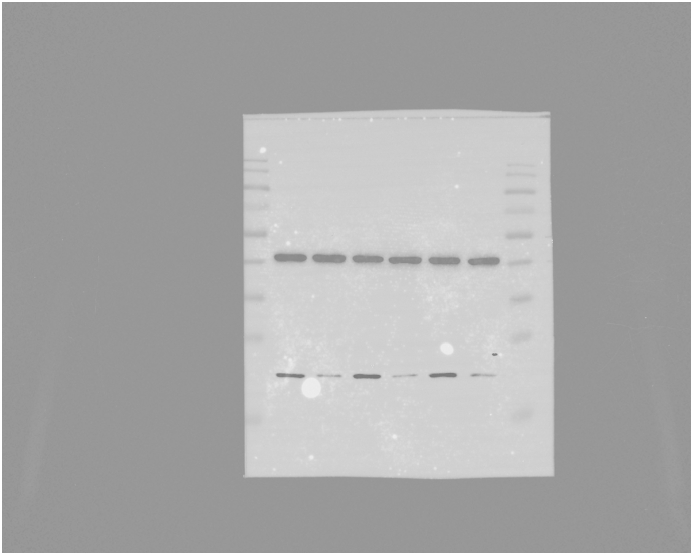


**figs3:**
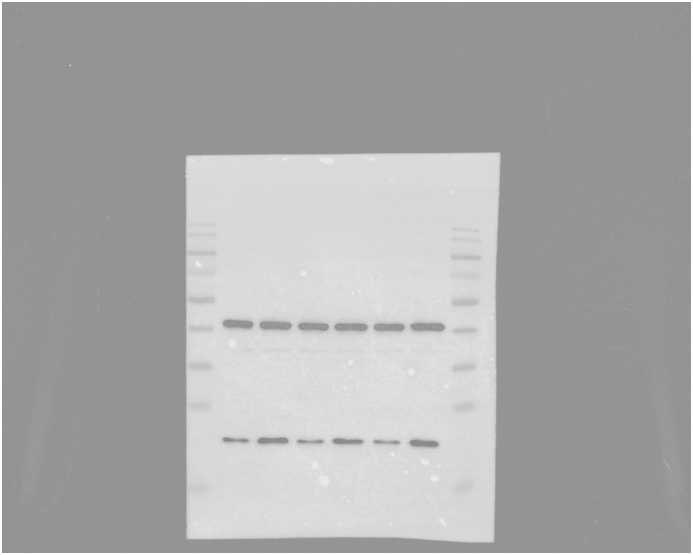


**figs4:**
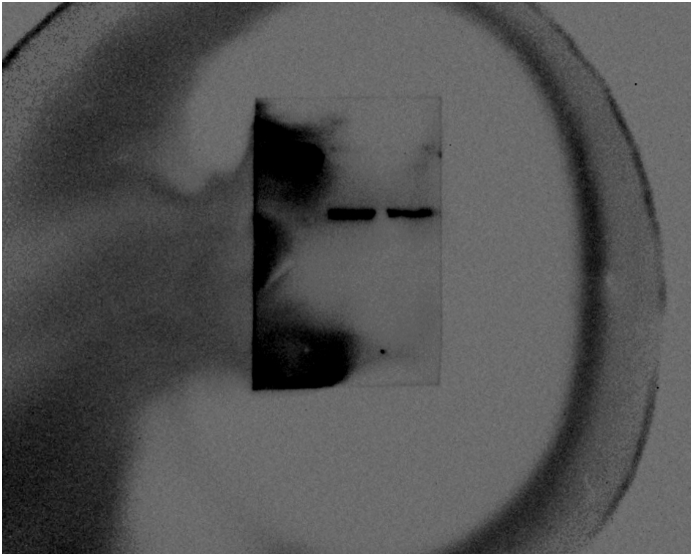


**figs5:**
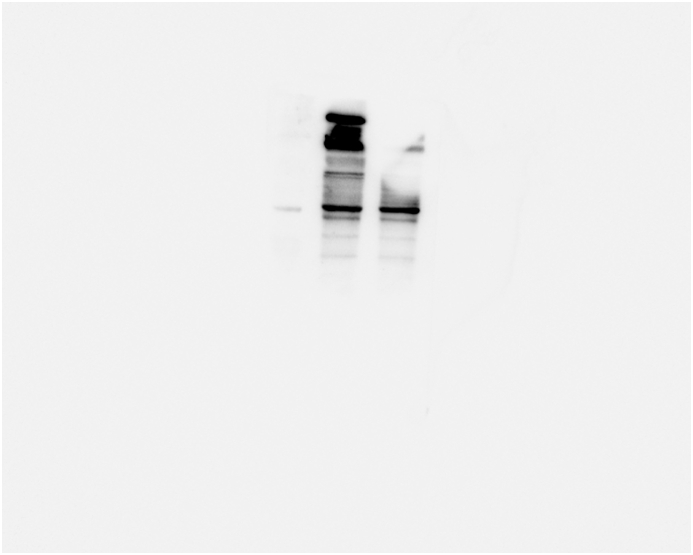


**figs6:**
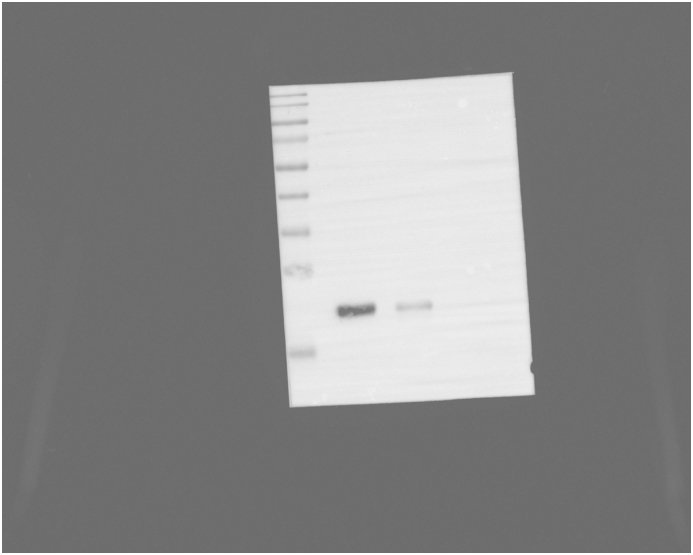


**figs7:**
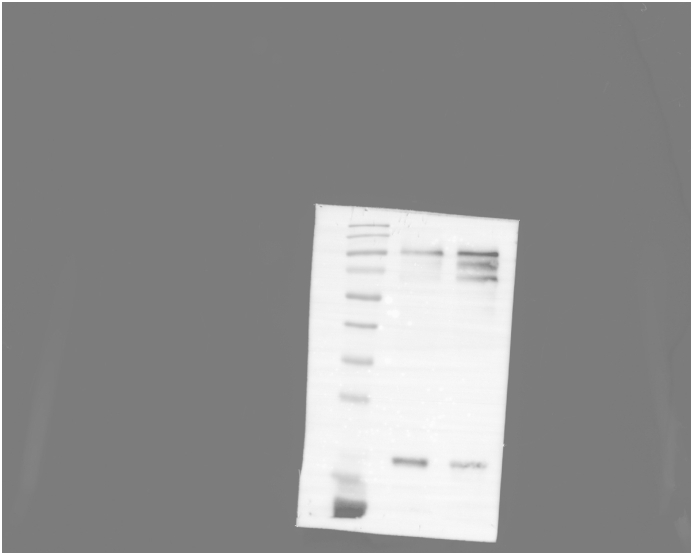


**figs8:**
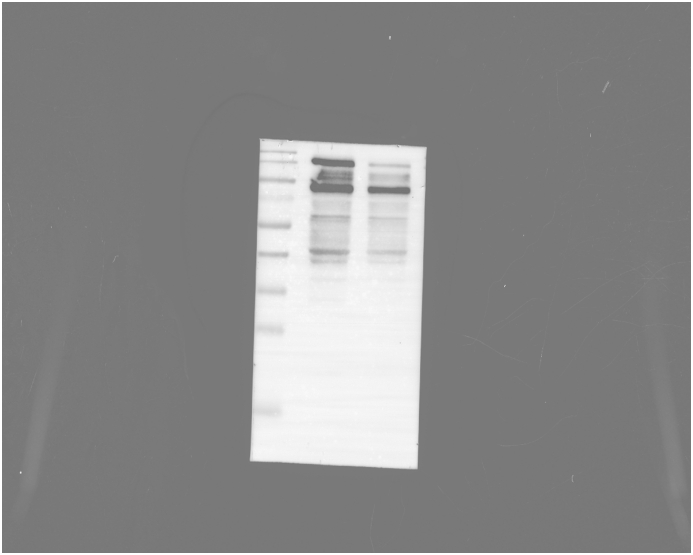


**figs9:**
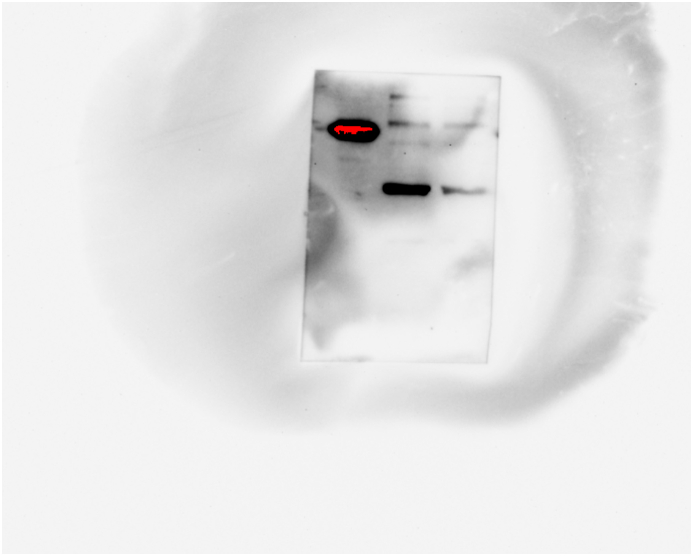


**figs10:**
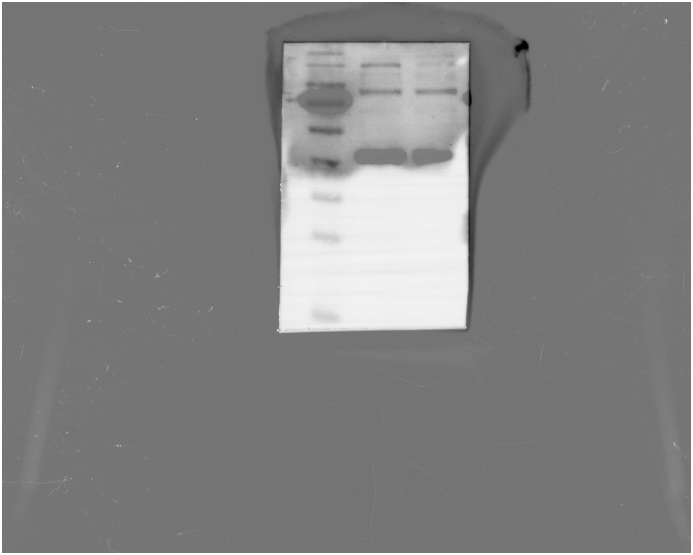


**figs11:**
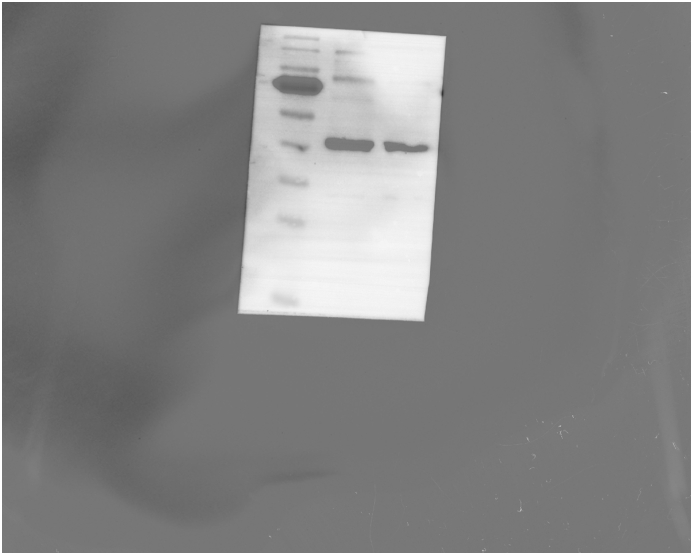


**figs12:**
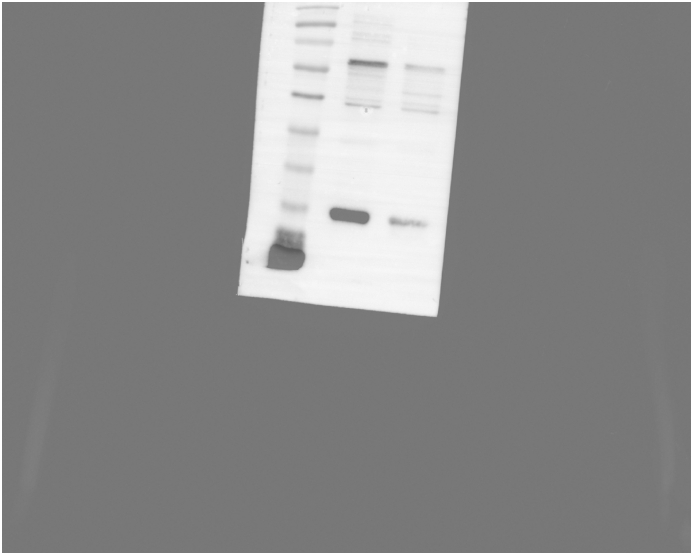


**figs13:**
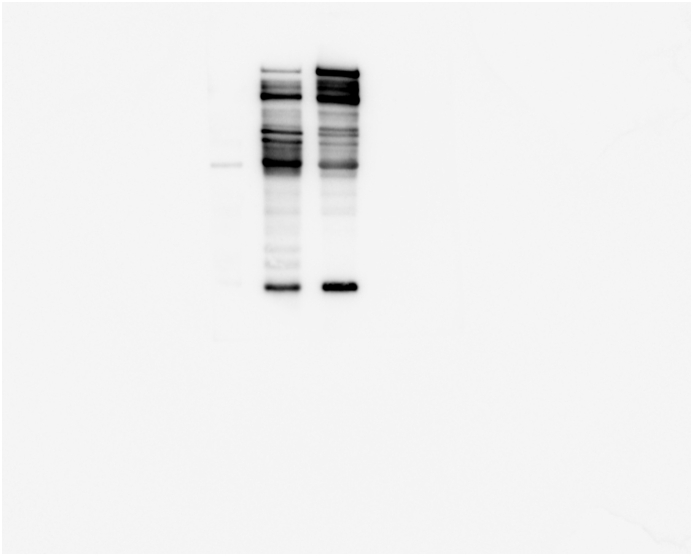


**figs14:**
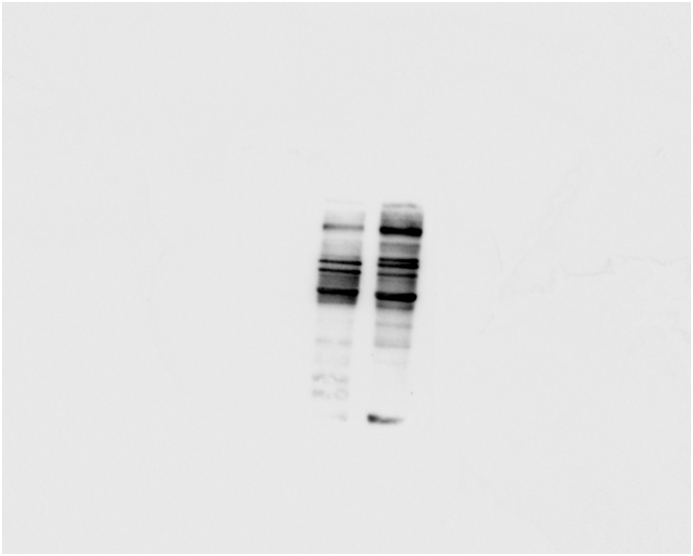


**figs15:**
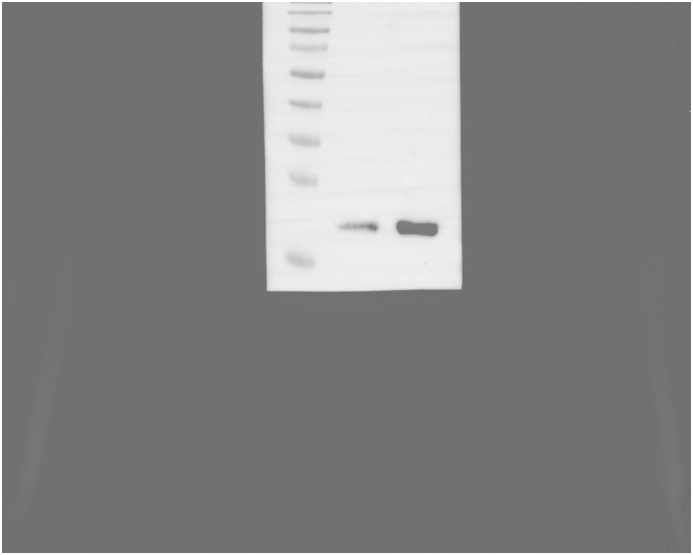


**figs16:**
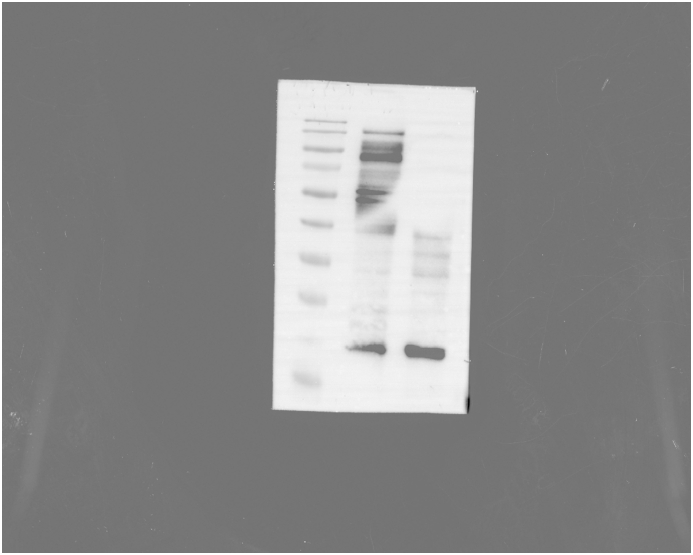


**figs17:**
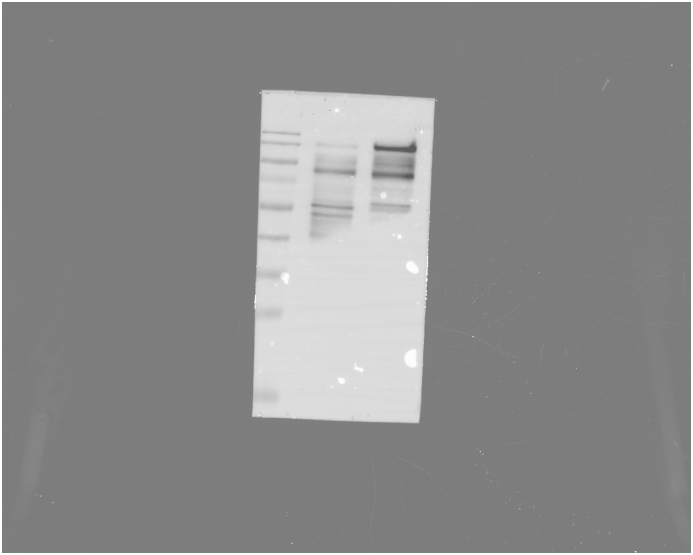


**figs18:**
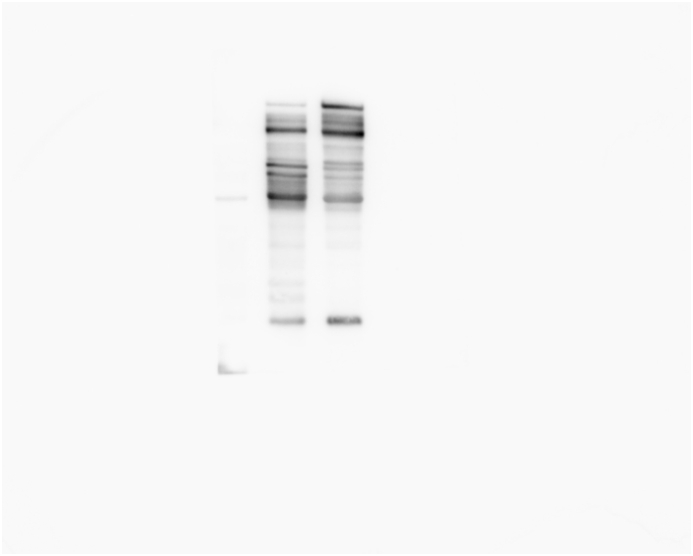


**figs19:**
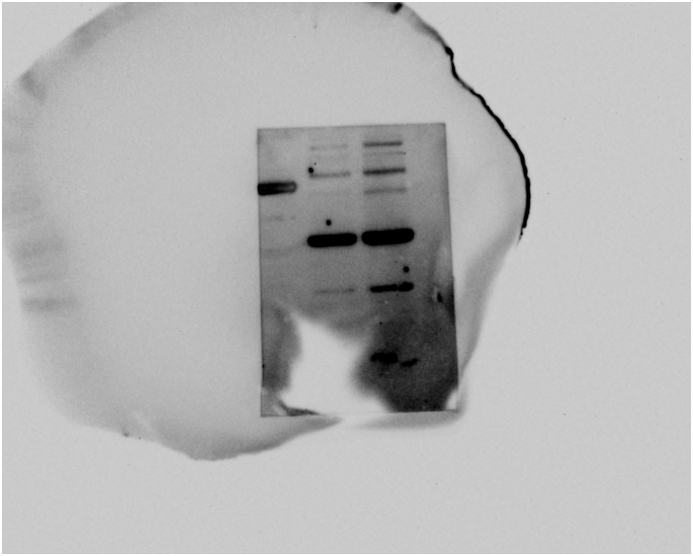


**figs20:**
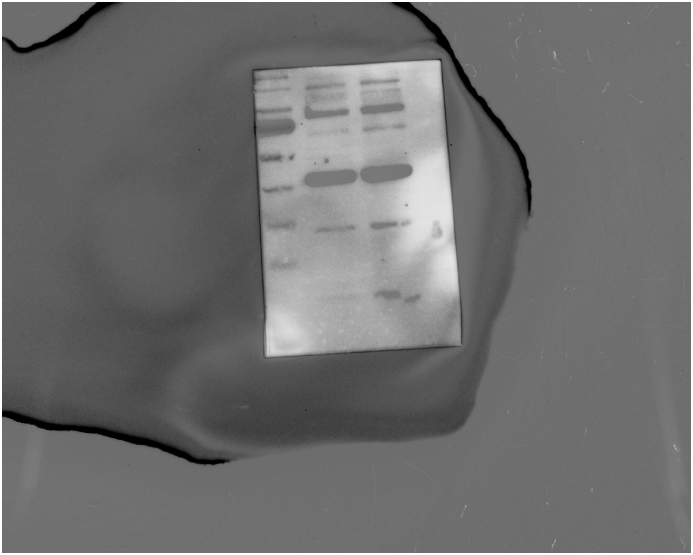


**figs21:**
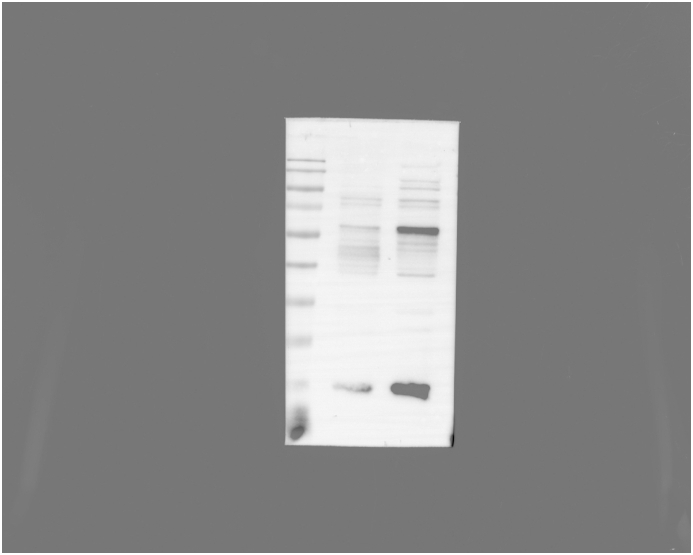


**figs22:**
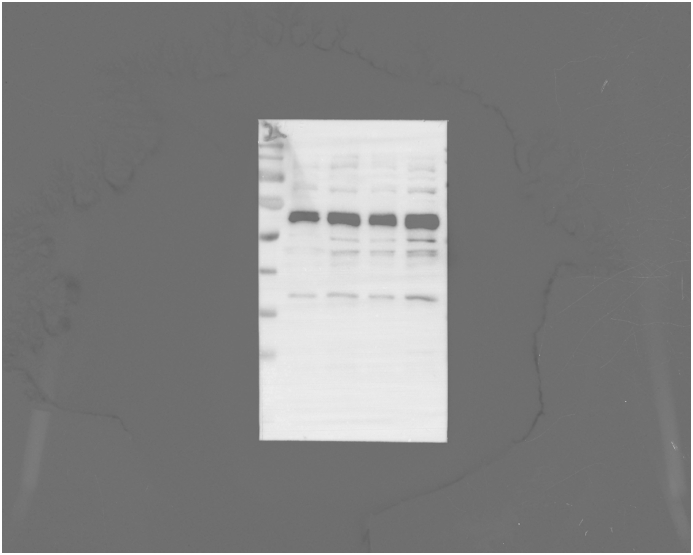


**figs23:**
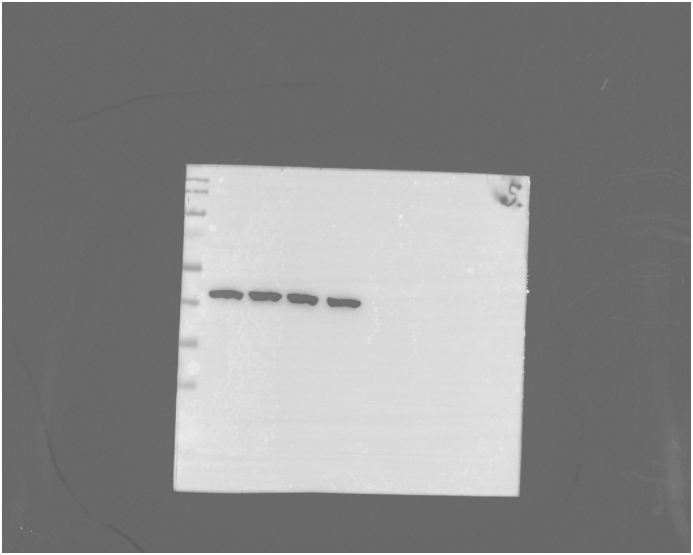


**figs24:**
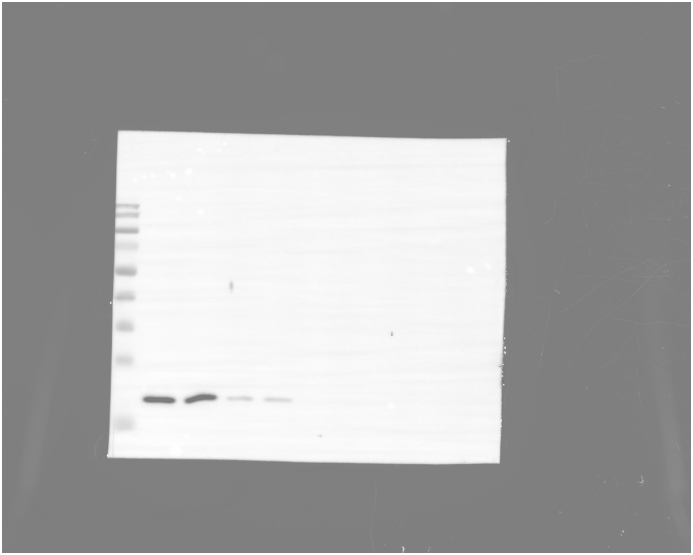


**figs25:**
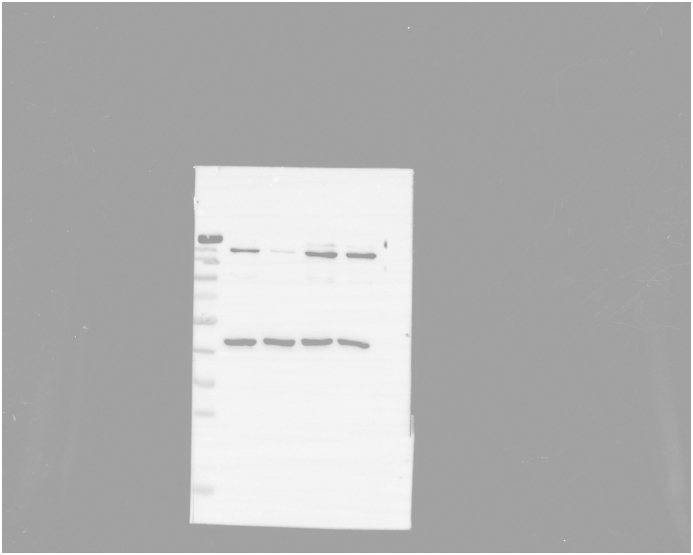


**figs26:**
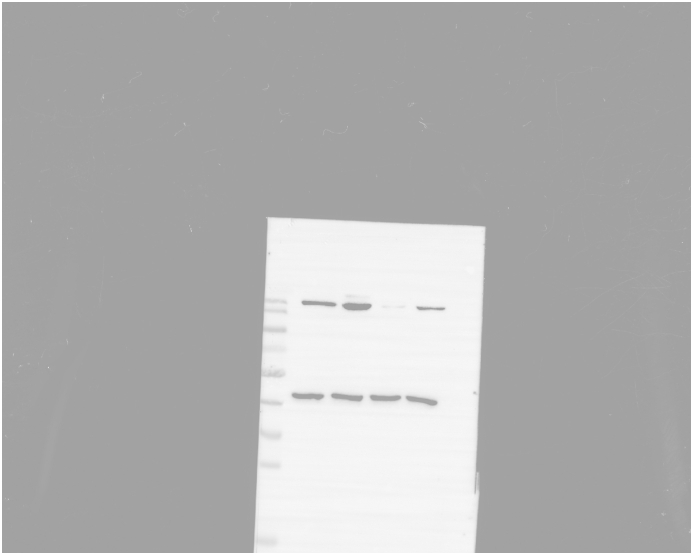


**figs27:**
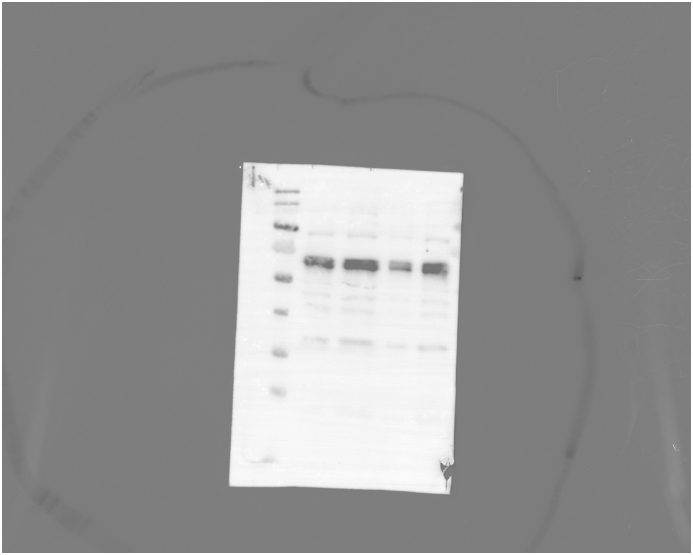


**figs28:**
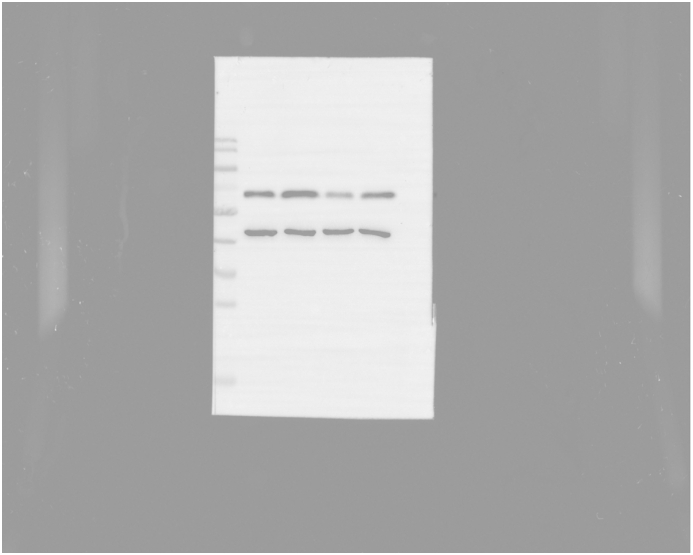


**figs29:**
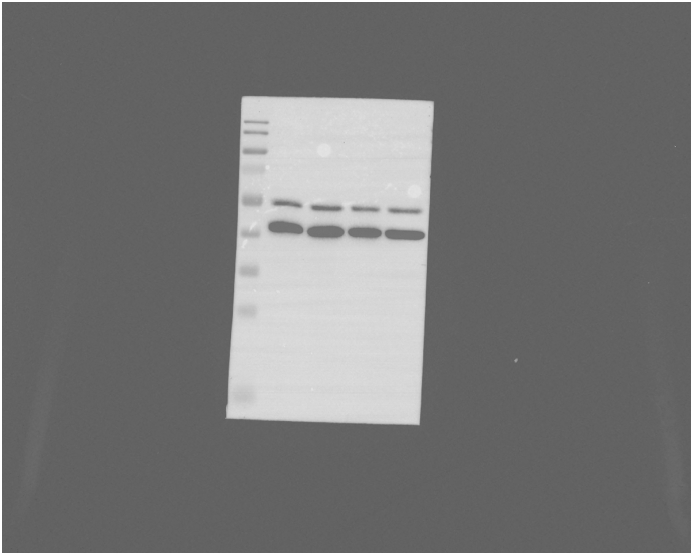


**figs30:**
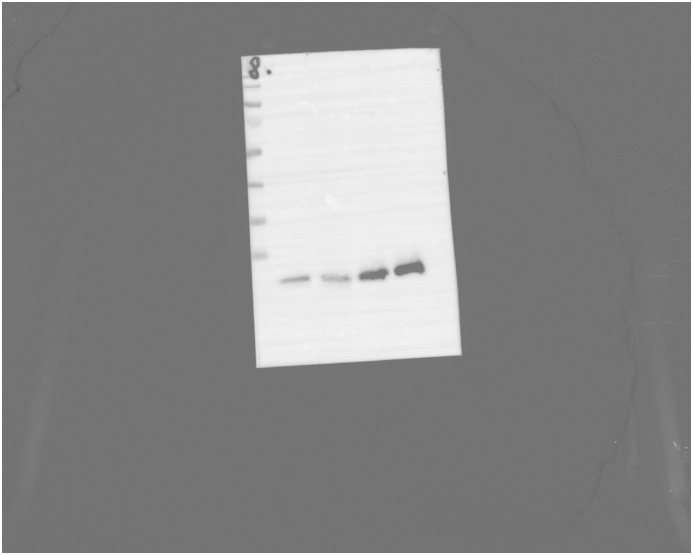


**figs31:**
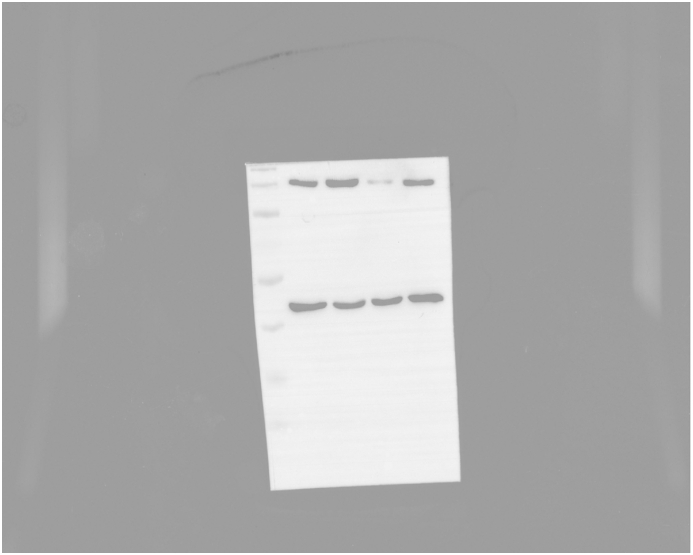


**figs32:**
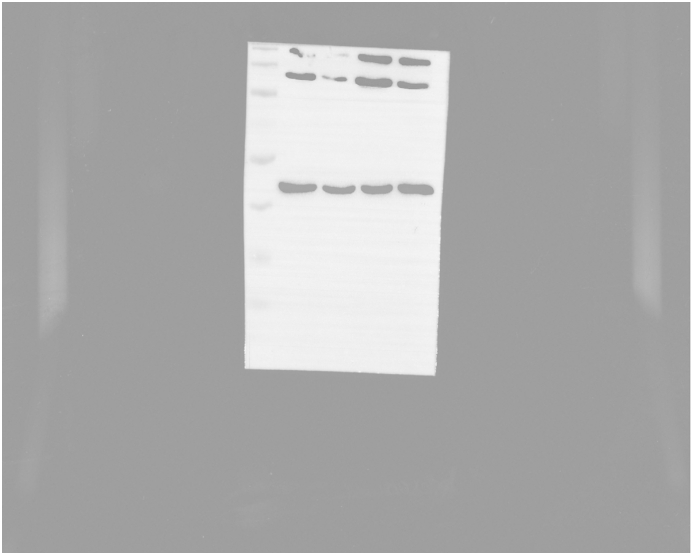


**figs33:**
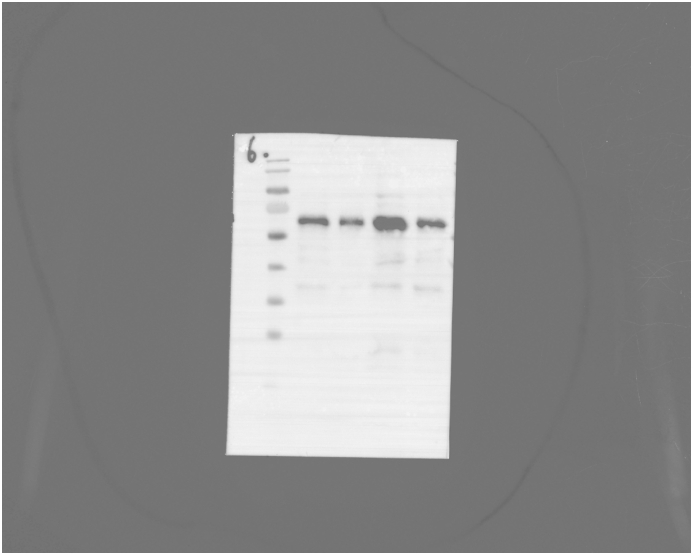


**figs34:**
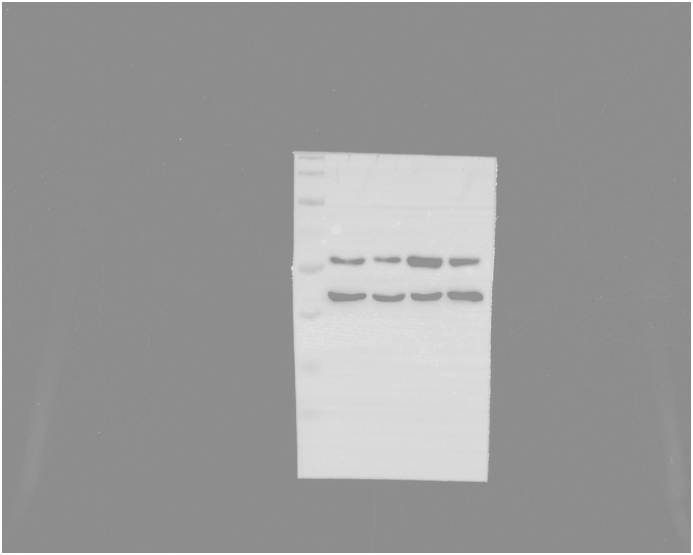


**figs35:**
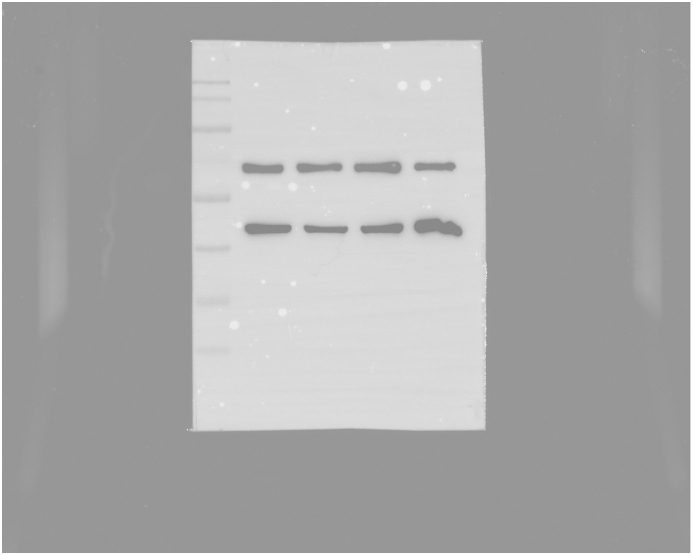


**figs36:**
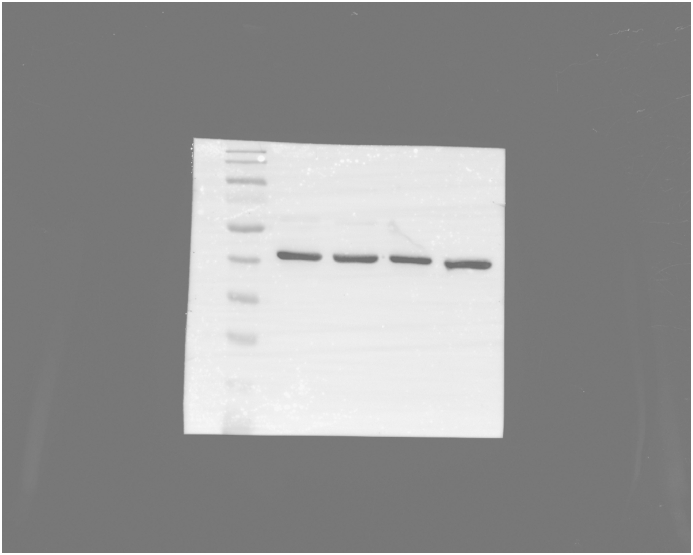


**figs37:**
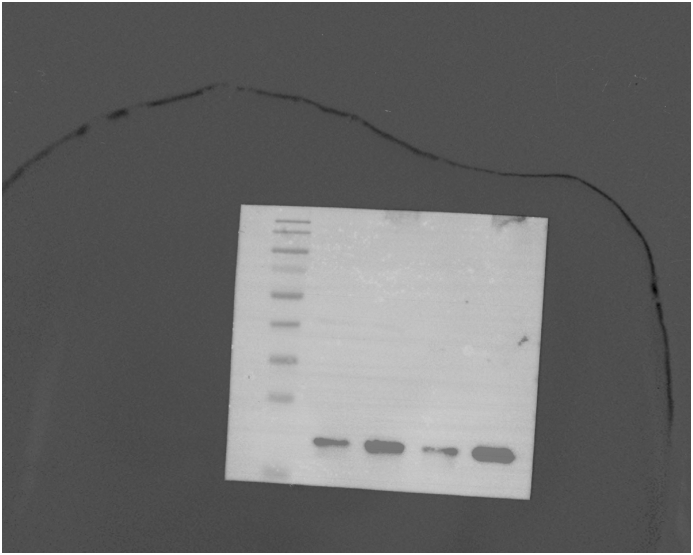


**figs38:**
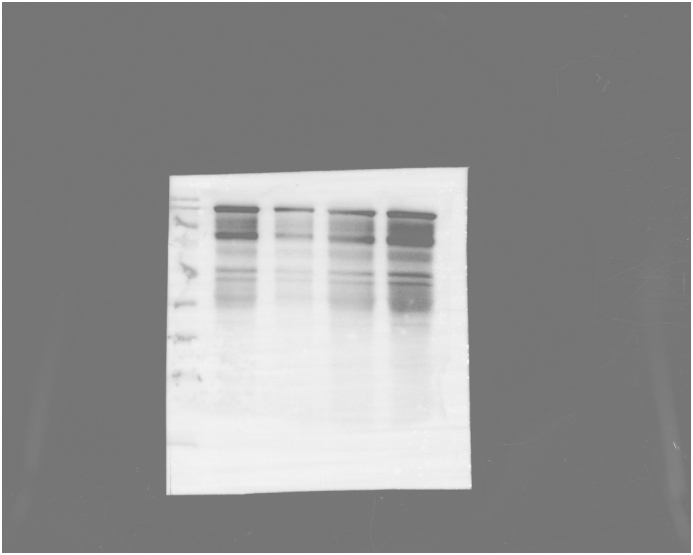


**figs39:**
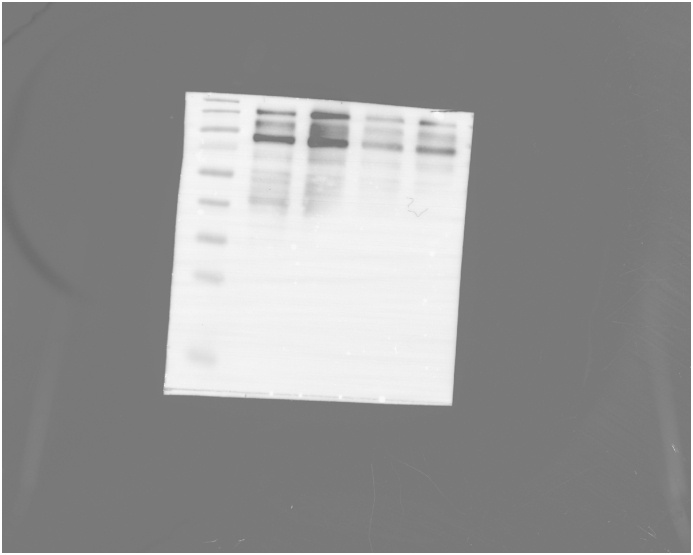


**figs40:**
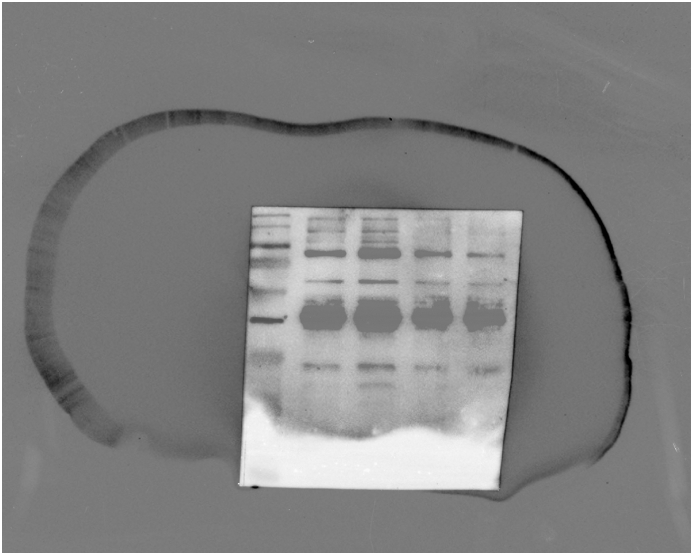


**figs41:**
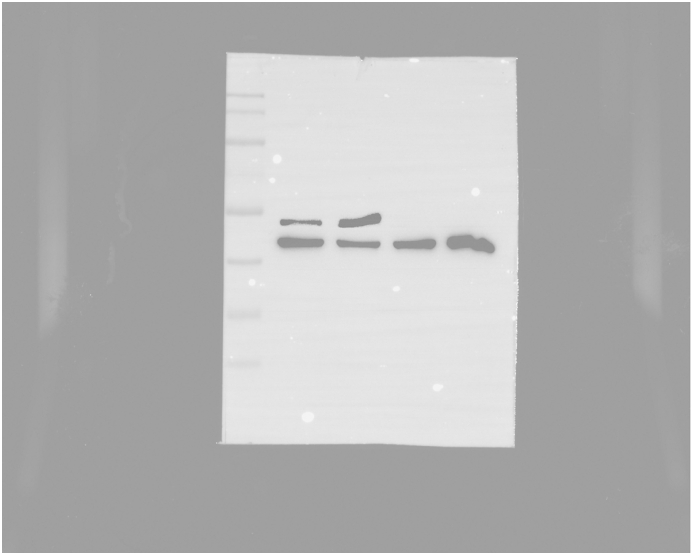


**figs42:**
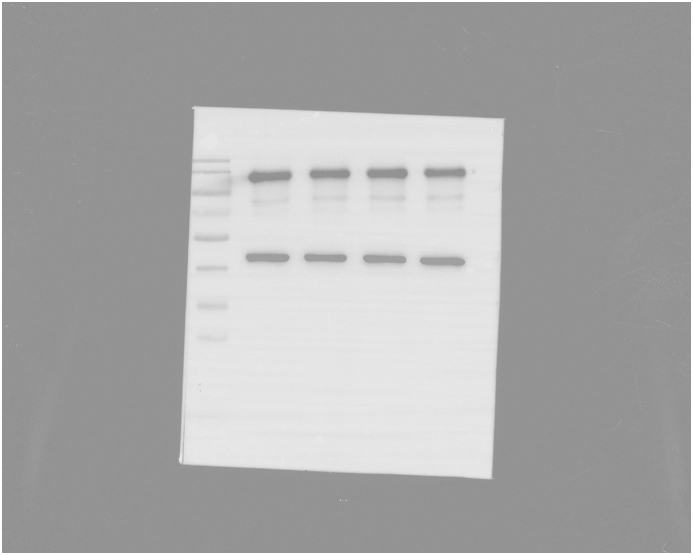


**figs43:**